# Does playing a wind instrument influence tooth position and facial morphology? Systematic review and meta-analysis

**DOI:** 10.1007/s00056-020-00223-9

**Published:** 2020-05-07

**Authors:** F. N. van der Weijden, R. B. Kuitert, F. Lobbezoo, C. Valkenburg, G. A. van der Weijden, D. E. Slot

**Affiliations:** 1 Department of Orthodontics, Academic Centre for Dentistry Amsterdam (ACTA), University of Amsterdam and Vrije Universiteit Amsterdam, Gustav Mahlerlaan 3004, 1081 LA Amsterdam, The Netherlands; 2 Department of Oral Kinesiology, Academic Centre for Dentistry Amsterdam (ACTA), University of Amsterdam and Vrije Universiteit Amsterdam, Amsterdam, The Netherlands; 3 Department of Periodontology, Academic Centre for Dentistry Amsterdam (ACTA), University of Amsterdam and Vrije Universiteit Amsterdam, Amsterdam, The Netherlands

**Keywords:** Music, Overjet, Arch width, Facial height, Lips, Musik, Overjet, Kieferbogenbreite, Gesichtshöhe, Lippen

## Abstract

**Purpose:**

To systematically search the scientific literature concerning the influence of playing a wind instrument on tooth position and/or facial morphology.

**Methods:**

The PubMed, EMBASE and Cochrane databases were searched up to September 2019. Orthodontic journals were hand searched and grey literature was sought via Google Scholar. Observational studies and (randomized) controlled clinical trials that assessed tooth position and/or facial morphology by profile cephalograms, dental casts or clinical examination were included. The potential risk of bias was assessed. Data from wind instrument players and controls were extracted. Descriptive analysis and meta-analysis were performed.

**Results:**

In total, 10 eligible studies with a cross-sectional (*n* = 7) or longitudinal design (*n* = 3) and an estimated low to serious risk of bias were included. Sample sizes ranged from 36 to 170 participants, varying from children to professional musicians. Descriptive analysis indicated that adults playing a single-reed instrument may have a larger overjet than controls. Playing a brass instrument might be associated with an increase in maxillary and mandibular intermolar width among children. Longitudinal data showed less increase in anterior facial height among brass and single-reed players between the age of 6 and 15. Children playing a wind instrument showed thicker lips than controls. Meta-analysis revealed that after a follow-up of 6 months to 3 years, children playing brass instruments had a significant reduction in overjet as compared to controls. The magnitude of the effect was of questionable clinical relevance and the generalizability was limited.

**Conclusions:**

Playing a wind instrument can influence tooth position and facial morphology in both children and adults. Aspects that stand out are overjet, arch width, facial divergence/convergence and lip thickness. However, evidence was sparse and the strength of the premise emerging from this review was graded to be “very low”.

**Electronic supplementary material:**

The online version of this article (10.1007/s00056-020-00223-9) contains supplementary material, which is available to authorized users.

## Abstract (de)

**Ziel:**

Systematische Suche nach wissenschaftlicher Literatur zum Einfluss des Spielens eines Blasinstruments auf die Zahnstellung und/oder die Gesichtsmorphologie.

**Methoden:**

Die Datenbanken PubMed, Embase und Cochrane wurden bis September 2019 durchsucht. Darüber hinaus wurden die wichtigsten kieferorthopädischen Zeitschriften per Hand und Google Scholar nach grauer Literatur durchsucht. Thematisch passende Studien wurden kritisch beurteilt und analysiert. Beobachtungsstudien und (randomisierte) kontrollierte klinische Studien, in denen die Zahnstellung und/oder die Gesichtsmorphologie anhand von Fernröntgenseitbild, Kiefermodellen bzw. klinischer Untersuchung beurteilt wurden, wurden einbezogen. Das potenzielle Bias-Risiko wurde bewertet. Daten von Blasinstrumentenspielern und Kontrollen wurden extrahiert, sowohl eine deskriptive Analyse als auch eine Metaanalyse wurden durchgeführt.

**Ergebnisse:**

Die Suche führte zu 10 geeigneten Studien, 7 Querschnitts- und 3 Längsschnittstudien mit einem geschätzten geringem bis erheblichem Bias-Risiko. Die Fallzahl reichte von 36 bis 170 Teilnehmern und die Merkmale der Studiengruppe variierten von Kindern bis hin zu professionellen Musikern. Die deskriptive Analyse zeigte, dass Erwachsene, die Einzelrohrblattinstrumente spielen, einen größeren Overjet haben können als Kontrollen. Das Spielen eines Blechblasinstruments steht bei Kindern möglicherweise in Zusammenhang mit einer Zunahme der intermolaren Breite im Ober- und Unterkiefer. Langzeitdaten zeigten eine geringere Zunahme der anterioren Gesichtshöhe bei Blechbläsern und Einzelrohrblattspielern im Alter zwischen 6 und 15 Jahren. Kinder, die ein Blasinstrument spielen, hatten im Vergleich zu den Kontrollen vollere Lippen. Die Metaanalyse zeigte, dass Kinder, die ein Blasinstrument spielen, bei einer Nachuntersuchungsdauer von 6 Monaten bis 3 Jahren eine signifikante Verkleinerung des Overjet im Vergleich zu den Kontrollen hatten. Das Ausmaß des Effekts war von fraglicher klinischer Relevanz, und die Generalisierbarkeit war begrenzt.

**Schlussfolgerung:**

Das Spielen eines Blasinstruments kann Zahnstellung und Gesichtsmorphologie bei Kindern und bei Erwachsenen beeinflussen. Hervorzuheben sind hierbei Overjet, Kieferbogenbreite, faziale Divergenz/Konvergenz und Lippendicke. Allerdings war die Evidenz spärlich und die Stärke der aus dieser Untersuchung hervorgehenden Prämisse wurde als „sehr gering“ eingestuft.

## Introduction

**Fig. 1:**
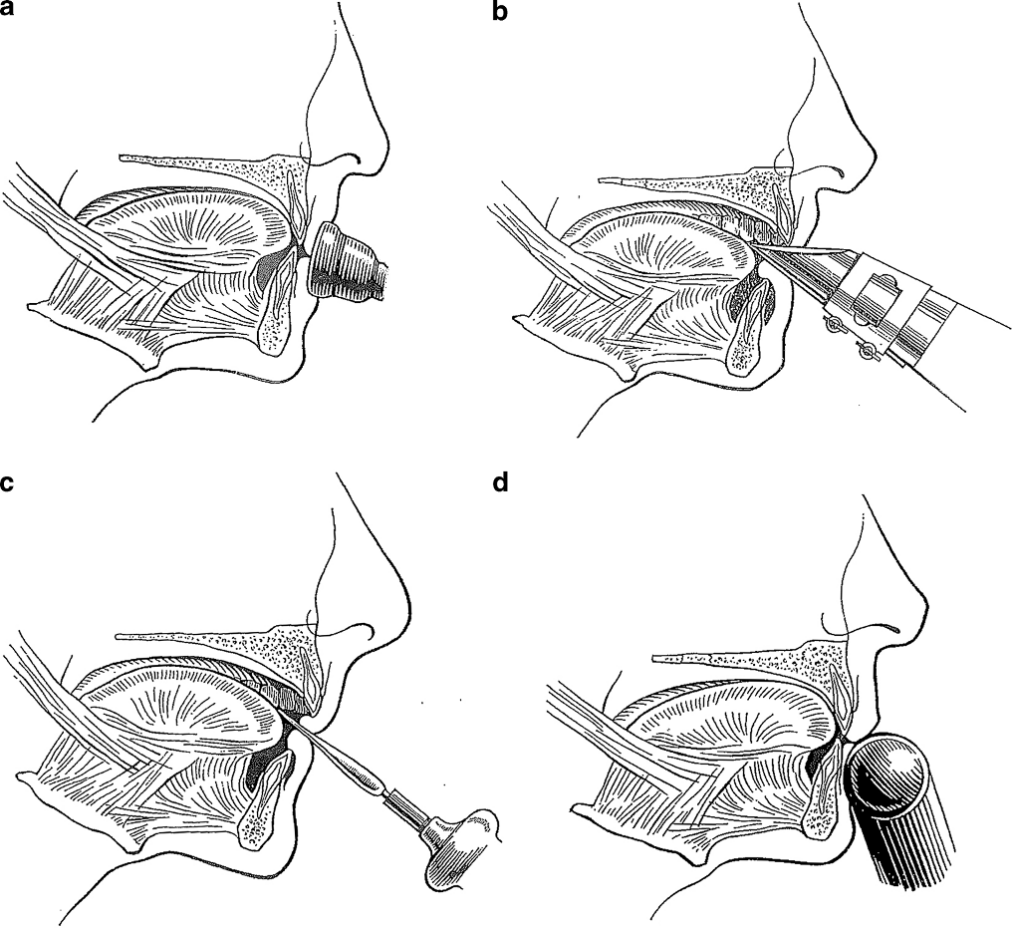
Schematic representation of how different types of wind instruments are held in, between, or against the mouth: **a** brass, **b** single-reed, **c** double-reed, **d** flute. (Reprinted with permission from [27])

The proposition that wind instruments have an influence on orofacial aspects and may enhance treatment of malocclusions and stimulate development towards a normal condition of the facial musculature was first described by Strayer [34]. He was an orthodontist and professional bassoonist and classified wind instruments according to the types of mouthpieces (Fig. 1). Different mouthpieces on wind instruments each require a specific technique to form “embouchure” [39], which may in turn provide different forces on the teeth and jaws. This “embouchure” concerns the whole complex of anatomical structures around the mouth and the way these structures are involved in playing the wind instrument [3].

Brass instruments (e.g., trumpet, trombone, horn, tuba) are placed outside the mouth by pressing the bowl-like mouthpiece against the upper and lower lip. Both upper and lower anterior teeth provide support for the lips. Depending on the height of the tone, the lips are pulled tight and set in vibrato ([3, 39]; Fig. 1a). When playing single-reed instruments (e.g., clarinet, saxophone) a wedge-shaped mouthpiece, to which a reed is attached at the underside, is placed partly in the mouth and between the lips. The maxillary incisors rest on the sloping upper surface of the mouthpiece, while the lower lip is placed between the lower surface of the reed and the mandibular incisal edges (single-lip embouchure) (Fig. 1b; [3, 39]). Double-reed instruments (e.g., oboe, bassoon) have a mouthpiece made from two bamboo reeds bound together with a cord. The two reeds are placed in the mouth, between the upper and lower lips, which cover the underlying incisal edges (double-lip embouchure) ([3, 39]; Fig. 1c). When playing the flute and piccolo, the mouthpiece is held against the lower lip, whereby the lower anterior teeth serve as a support. The upper lip is pushed downward to form a small slit-shaped opening between the lower and upper lip, through which air is directed towards the opposite rim of the blowhole. The embouchure of the flute is partly controlled by the position of the flute in relation to the upper lip. This is done by a rotation movement of the flute in the plica mentalis in combination with alternating protrusion and retrusion of the mandible ([3, 39]; Fig. 1d).

According to Proffit’s equilibrium theory [29], the position of teeth depends on forces exerted from the tongue and lips, from the dental occlusion, and from the periodontal membrane as well as from habits like thumb sucking. Theoretically, playing a wind instrument exerts external forces comparable to oral parafunctional habits and hence might result in improvement or deterioration of (mal)occlusion [13]. The forces produced by playing a musical instrument are larger than forces produced by average muscle contraction and approach pressure levels associated with maximum lip–muscle effort [9]. Furthermore, playing wind instruments requires increased intraoral air pressure. It has been shown that the mode of air pressure and the orofacial muscle activity may separately influence dentofacial morphology [5]. Taken together, this gives credence to the supposition that “embouchure” pressure should be given consideration in orthodontic rationale.

Therefore, the aim of this systematic review was to comprehensively search the scientific literature in order to identify, critically appraise, analyze, and synthesize studies concerning the influence of playing of wind instrument on tooth position and facial morphology.

## Methods

### Protocol

The recommendations for strengthening the reporting were followed in accordance with the Cochrane Handbook [17], Preferred Reporting Items for Systematic Reviews and Meta-Analyses (PRISMA) [28], MOOSE Guidelines for Meta-Analyses and Systematic Reviews of Observational Studies [35] and the PRISMA extension for abstracts [2]. The protocol for the review method was developed a priori, following initial discussions among members of the research team.

### Focused question (PICOS)

The question to be answered was the following: What is the difference in tooth position and/or facial morphology between people who play a wind instrument and those who do not, as found in observational studies and (randomized) controlled clinical trials?

### Eligibility criteria

The following criteria were imposed for inclusion in the systematic review:

- Observational studies and (randomized) controlled clinical trials describing the effect of playing a wind instrument on tooth position and facial morphology.
- Studies comparing people who play a wind instrument and those who do not.
- Any study involving cephalometrics, dental casts, or clinical outcome on tooth position and facial morphology related to paying wind instruments.

### Information sources and search

The PubMed-MEDLINE, EMBASE and Cochrane-CENTRAL databases were searched from initiation up to September 2019 (F.N.W., D.E.S.). The search strategy is listed in Table 1. Furthermore, several orthodontic journals (*American Journal of Orthodontics and Dentofacial Orthopedics* [Volume 1, Issue 1, January 1915–Volume 156, Issue 3, September 2019]; *European Journal of Orthodontics *[Volume 1, Issue 1, January 1979–Volume 41, Issue 4, August 2019]; *Angle Orthodontist* [Volume 1, Issue 1, January 1931–Volume 89, Issue 5, September 2019]; (British) *Journal of Orthodontics* [Volume 1, Issue 1, 1973–Volume 46, Issue 2, June 2019]; *Journal of Orofacial Orthopedics* [Volume 1, Issue 1, January 1931–Volume 80, Issue 5, September 2019]; *Seminars in Orthodontics* [Volume 1, Issue 1, January 1979–Volume 25, Issue 2, June 2019]; *Transactions of the European Orthodontic Society* [1955–1979]) were searched for papers older than the inception date of PubMed or papers that might have been improperly indexed in PubMed [23], using the search engine as provided by these journals (R.B.K., F.N.W.) or hand searched (R.B.K.). Also grey literature was searched via Google Scholar (using various combinations of the following keywords: tooth position, malocclusion, wind instrument, musical instrument, performance). In addition, the reference lists of all selected studies were hand searched for additional relevant articles (F.N.W., R.B.K.).

**Table 1:** Search strategy for PubMed. The search strategy was customized according to the database being searched

(<“Malocclusion”[Mesh] OR malocclusion> OR <“Dental Occlusion”[Mesh] OR occlusion> OR <“Orthodontics”[Mesh] OR orthodontic*> OR <tooth AND position*> OR <“Face”[Mesh] OR facial OR orofacial OR oro-facial OR oral-facial>)	AND (<instrument AND {“Music”[Mesh] OR Music}> OR <wind AND instrument*>)

*The asterisk was used as a truncation symbol

### Study selection

Titles and abstracts of the studies obtained from the searches were screened independently by two reviewers (F.N.W., G.A.W.) and were categorized as definitely eligible, definitely not eligible, or questionable. No language restrictions were imposed. No attempt was made to blind the reviewers to the names of authors, institutions, or journals while making the assessment. If eligible aspects were present in the title, the paper was selected for further reading. If none of the eligible aspects were mentioned in the title, the abstract was read in detail to screen for suitability. Papers that could potentially meet the inclusion criteria were obtained and read in detail by the two reviewers (F.N.W., G.A.W.). Disagreements in the screening and selection process concerning eligibility were resolved by consensus or, if disagreement persisted, by arbitration through a third reviewer (R.B.K.). The papers that fulfilled all of the inclusion criteria were processed for data extraction.

### Data collection process, summary measures and synthesis of results

When provided, information about the characteristics of the study sample population, assessed parameters, and outcomes were extracted from all the studies by two authors (F.N.W., R.B.K.). In order to provide a summary overview of differences of tooth position and facial morphology between wind instrument players and the control group, the outcomes of the selected studies were categorized (F.N.W., G.A.W.). If a statistical analysis was lacking in the original paper but the data provided (sample size, mean and standard deviation [SD] per group of interest) did allow this, *p*-values were calculated by one of the authors of this review (F.N.W.) with the help of a statistician.

If feasible, the data from the included studies were synthesized into a meta-analysis (D.E.S., F.N.W., C.V.). In studies consisting of multiple comparisons and data from one particular group compared with more than one other group, the number of subjects (*n*) in the group was divided by the number of comparisons. A meta-analysis was only performed if at least two studies could be included. The difference of means between test and control was calculated using a random effects model. The goal was to estimate the mean effect in a range of studies where the overall estimate is not overly influenced by any one of them [4]. In addition to the difference of means (DiffM) and 95% confidence intervals (95% CI), we calculated 95% prediction intervals. The advantage of prediction intervals is that they reflect the variation in treatment effects across different settings, including what effect is to be expected in future patients [19]. Computations for the meta-analysis were performed using R (https://project.org) with the packages meta [32] and metafor [38].

As planned a priori, relative to the type of wind instrument, a subgroup analysis was conducted. If possible formal testing for publication bias was performed as proposed by Egger et al. [8]. Based on the descriptive and meta-analysis a synthesis of results was given.

### Potential risk of bias

For the individual studies two reviewers (F.N.W., G.A.W.) scored the potential risk of bias of the included studies, using a comprehensive combination of the Critical Appraisal Checklist for Analytical Cross Sectional Studies, developed by the Joanna Briggs Institute [20], the Newcastle Ottawa scale adapted for cross-sectional studies [18], and the ROBINS- I tool [7] as earlier reported by Van der Weijden et al. [37].

### Assessment of heterogeneity

Across studies several factors used to evaluate the clinical and methodological heterogeneity of the characteristics of the different studies were as follows: study design, participants, and variables used to assess tooth position and facial morphology.

As part of the meta-analysis, heterogeneity was statistically tested by the χ^2^ test and the I^2^ statistic. A χ^2^ test resulting in a *p* < 0.1 was considered an indication of significant statistical heterogeneity. As an approximate guide to assessing the possible magnitude of inconsistency across studies, an I^2^ statistic of 0–40% was interpreted to indicate unimportant levels of heterogeneity. An I^2^ statistic of 30–60% may represent moderate heterogeneity an I^2^ statistic of 50–90% may represent substantial heterogeneity, and a I^2^ statistic of greater than 75% was interpreted to indicate considerable heterogeneity [31]. Considerable heterogeneity was assessed with sensitivity analysis to assess the effect modification.

### Rating the certainty and quality of the evidence

The Grading of Recommendations Assessment, Development and Evaluation (GRADE) system was used, as proposed by the GRADE working group, to appraise the evidence emerging from this review. Two reviewers (F.N.W., G.A.W.) rated the strength of the evidence according to the following aspects: estimated potential risk of bias, consistency of results, directness of evidence, precision and publication bias, and magnitude of the effect [12, 15]. Any disagreement between the two reviewers was resolved after additional discussion.

## Results

### Study selection

**Fig. 2:**
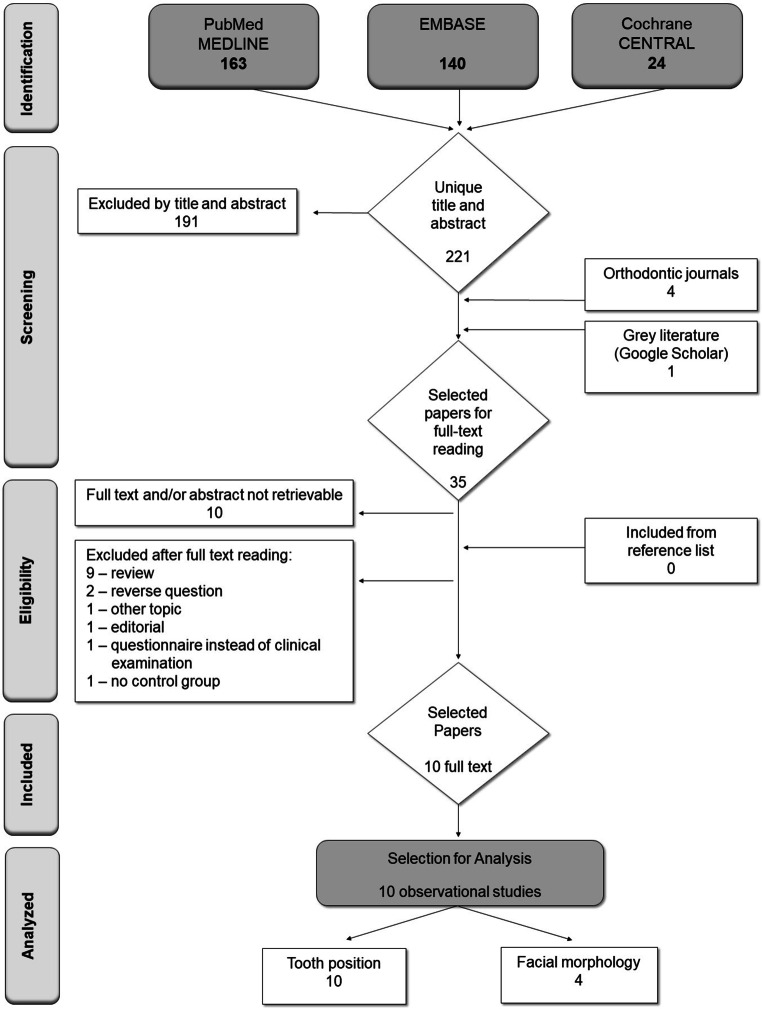
Flowchart of search and selection

The searches on PubMed-MEDLINE, EMABSE and Cochrane-CENTRAL resulted in 221 unique papers (Fig. 2). Screening of the titles and abstracts resulted in 30 potentially suitable papers. The full text of 10 papers was not retrievable. For 20 papers, the full texts were obtained and read in full. Of these, nine [1, 5, 10, 13, 14, 16, 25, 30, 32] met the eligibility criteria. Searching the main orthodontic journals further revealed four potentially suitable papers, of which none appeared eligible after full text reading. Google scholar yielded one suitable paper [6]. Screening the reference lists of the ten selected full text papers resulted in no additional papers. Thus, a total of 10 papers (Fig. 2) were included in this systematic review.

### Study characteristics

The study characteristics of the included papers are listed in Table 2. Across the 10 included studies there was a large heterogeneity in study design and type of participants. Seven of the eligible papers had a cross-sectional study design, whereas three had a longitudinal design. Three originated from the USA, two from Switzerland, one from Japan, and the remainder from the UK, Norway, Nigeria, and Kenya. The sample sizes ranged from 36 to 170 participants. The population characteristics varied from children to professional musicians.

**Table 2:** Study characteristics of the included papers

Authors (Year of publication) Geographic location	Study design Examination Estimated potential risk of bias (Online Appendix 1)	Number of participants	Population Age Gender	Conclusions of the original authors
Adeyemi et al. (2019) [1] Nigeria	– Cross-sectional – Dental casts – Low	100 – 50 wind players (36 trumpet, 4 trombone, 4 clarinet, 6 saxophone) – 50 non-wind players	– Wind players who had been playing their instrument for a minimum of 2 years (mean 9.26 ± 6.21, range 2–25) – 28.05 ± 7.56 years, range 18–45 – 100 male	Playing a wind musical instrument did not significantly affect the occlusal characteristics except the maxillary anterior segment alignment. Increased irregularity of the maxillary anterior segment was significantly pronounced in trumpet/trombone players
Brattström et al. (1989) [5] Norway	– Retrospective longitudinal, 9 years – Profile cephalograms, dental casts – Moderate	98 – 58 wind players (38 brass, 20 single-reed) – 40 non-musicians	– Children from school orchestras – Follow-up evaluations: 6, 9, 12, 15 years – 38 male, 60 female	Playing musical wind instruments influenced the dentofacial skeleton giving a widening of the dental arch and a reduction of the anterior facial height as a result of anterior rotation of the maxilla and the mandible
Bwire (2013) [6] Kenya	– Cross-sectional – Clinical examination – Moderate	103 – 63 wind players (36 brass, 21 single-reed, 6 flute) – 40 non-wind players (28 string, 8 percussion, 4 others)	– Musicians with mixed experience (66.7% 1–3 years; 28.6% 4–6 years; 4.8% ≥7 years) – 22.38 ± 5.68 years, range 14–40 – 73 male, 30 female	There was no difference in malocclusion patterns between wind and non-wind players, neither between players of different types of wind instruments
Fuhrimann et al. (1987) [10] Switzerland	– Cross-sectional – Profile cephalograms, dental casts – Moderate	36 – 12 trumpeters – 12 clarinetists – 12 control (dental students)	– Professional musicians – Mean 26 years ^c^, range 19–55 – 32 male, 4 female	There was no difference between the two groups or musicians, nor in comparison with a control group, regarding lip morphology and bite morphology
Grammatopoulos et al. (2012) [13] United Kingdom	– Cross-sectional – Dental casts – Moderate	170 – 111 wind players (32 large brass^a^, 42 small brass^b^, 37 single-reed) – 59 non-wind players (string and percussion)	– Professional musicians – Age: NR – Male/Female: NR	A wind instrument did not significantly affect anterior tooth position. However, playing brass instrument with a large cup-shaped mouthpiece, such as the trombone and tuba, might pose a small risk for the musician to develop a lingual crossbite
Gualtieri (1979) [14] USA	– Cross-sectional – Clinical examination – Serious	115 – 82 wind players (16 large brass^a^, 35 small brass^b^, 19 single-reed, 6 double-reed, 6 flute) – 33 controls (piano and percussion players, dental hygiene students, dental assistants)	– (Semi-) professional musicians – 18–61 years, with the majority being 18–25 years – 75 male, 40 female	Although there were some significant differences in the incidence of certain pertinent oral and facial entities between some wind-instrument musicians and their counterpart controls, among mature persons, there is no reason to categorically prohibit all persons with potential malocclusions from studying music
Herman (1981) [16] USA	– Prospective longitudinal, 2 years – Clinical examination, dental casts – Serious	Start of the study: 276 – 220 wind players – 56 controls End of the study: 127 – 91 wind players (21 brass, 41 single-reed, 4 double-reed, 25 flute) – 36 controls	– Beginning musicians – 11–13 years – Male/Female: NR	Statistically significant anterior tooth movements occurred in an overwhelming majority of the instrumentalists, while negligible movements were recorded for the controls over this period. The playing of the correct musical wind instrument can serve as an adjunct to the dentist or orthodontist in trying to accomplish certain tooth movements
Pang (1976) [25] USA	– Prospective longitudinal, 6 months – Dental casts – Moderate	Start of the study: 84 End of the study: 76 – 30 non-musicians – 46 wind players (19 brass, 21 single-reed, 2 double-reed, 4 flute^c^)	– Beginning musicians – Seventh graders – 37 male, 39 female ^c^	On a group basis, the class A musical wind instruments reduced the overjet. On a group basis, the class B musical wind instruments did not produce an overjet. On an individual basis, the effect of musical wind instruments is unpredictable and cannot be used as a substitute for orthodontics. The study should be carried out for longer than a 6-month period
Rindisbacher et al. (1990) [30] Switzerland	– Cross-sectional – Dental casts and profile cephalograms – Moderate	137 – 62 wind players (31 brass (12 trumpet, 10 trombone); 31 reed and flute (13 clarinet, 10 oboe or bassoon, 8 flute)) – 75 control (54 dental students, 21 university students)	– Professional musicians – Brass: mean 25 years, range 21–56 – Reed and flute: mean 27 years, range 18–44 – Controls: mean 24 years, range 20–33 – 129 male, 8 female	There was no or only minor influence on the face or dentition from playing wind instruments
Shimada (1978) [33] Japan	– Cross-sectional – Clinical examination, profile cephalograms regarding both hard and soft tissues, dental casts – Moderate	– 55 wind players (10 large brass^a^, 13 small brass^b^, 17 single-reed, 4 double-reed, 11 flute) – 20–60 nonspecified controls, regarded as normal/ideal	– Pupils that had been active members in the music clubs for longer than 2 years – Mean 16 years, range 13–18 – 46 male, 9 female	The effect attributable to the playing of wind instruments mainly manifested itself in the inclination angle of anteriors, widths of dental as well as alveolar basal arches, and in the depth of upper and lower lip region

*NR* not reported

^a^ Large brass = trombone, baritone, bass horn, tuba

^b^ Small brass = trumpet, bugle, French horn, alto horn

^c^ Calculated by the authors of this review based on the data presented in the selected paper

### Potential risk of bias

Potential confounding factors (see Online Appendix 1) were defined and assessed in all selected studies, but were analyzed in only three [1, 13, 25]. In only three studies [14, 16, 32], the sample was clearly defined and representative. The sample size was justified and satisfactory in only two studies [1, 13]. For all studies, except one [16], the outcome data of (nearly) all participants were available. The assessment of tooth position and facial morphology was measured in a valid and reliable way in all studies, except one [14]. This study did not report the assessment criteria. Four studies reported that the investigators were blinded [1, 13, 25] and/or calibrated [1, 6]. Three studies [14, 16, 25] did not (or only partly) used appropriate statistical analysis. Overall, the potential risk of bias of the included studies was estimated to be “low” for one [1], “serious” for two [14, 16] and “moderate” for the other seven studies. Because the ten papers provided heterogeneous outcome parameters, publication bias could not be assessed.

### Descriptive analysis

**Fig. 3:**
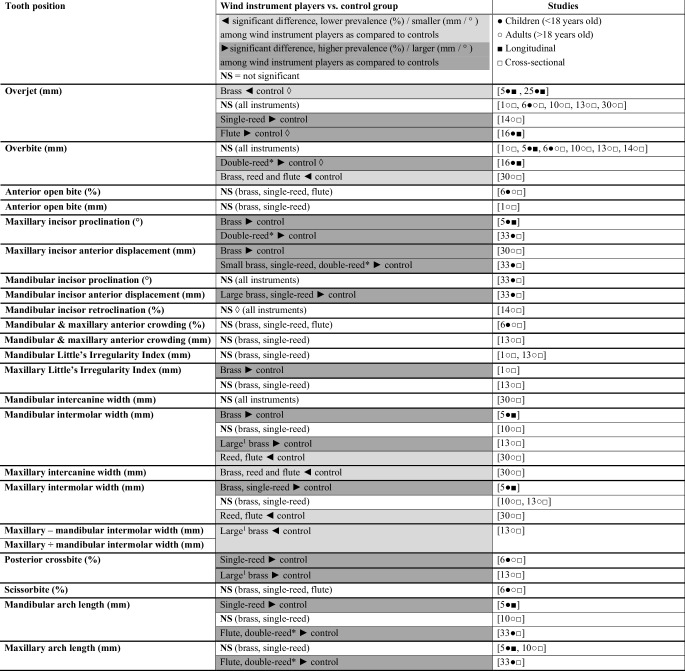
Descriptive overview of comparisons on tooth position according to the included studies. ^1^ Large brass: trombone, baritone, bass horn, tuba, ^2^ Small brass: Trumpet, bugle, French horn, alto horn; *Diamond *Calculated by the authors of this review based on the data presented in the selected paper. To calculated the standard deviation of the difference score after 2 years for the study by Herman [16], a correlation of 0.5 was used. *Asterisk* The double-reed group consisted of only 4 musicians, which makes the relevance of statistical significance questionable

**Fig. 4:**
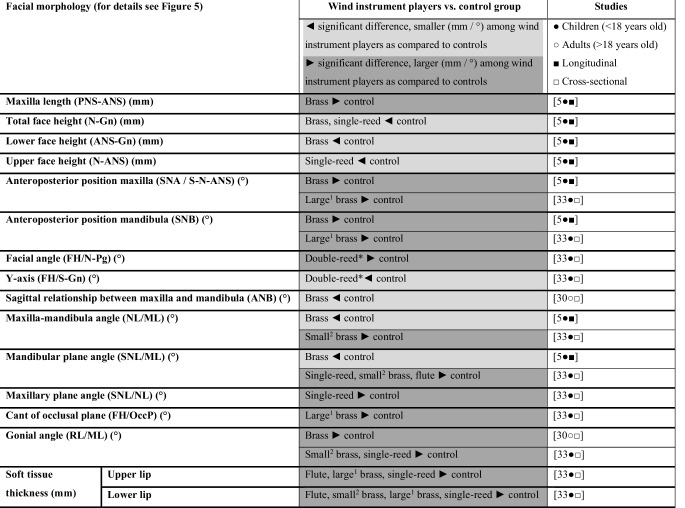
Descriptive overview of comparisons on facial morphology according to the included studies. ^1^ Large brass: trombone, baritone, bass horn, tuba; ^2^ Small brass: trumpet, bugle, French horn, alto horn; *Asterisk* The double-reed group consisted of only 4 musicians, which makes the relevance of statistical significance questionable

Descriptive data extracted from the included studies are presented in Figs. 3 and 4. These figures provide a summary overview of differences in features of tooth position and facial morphology between wind instrument players and control group.

### Meta-analysis

A meta-analysis was only feasible for the outcome parameter overjet. Three studies [5, 16, 25] examined overjet longitudinally among 11- to 15-year-old beginning brass and single-reed instrument players. The follow-up period was different for each study: 6 months [25], 2 years [16], or 3 years [5]. Baseline was considered the start of the study and end the finalization of the study. Whereas at baseline no significant differences were found, at the end of the follow-up period brass instrument players had a significant smaller overjet as compared to controls (−0.67 mm, 95% CI 95% [−1.02,−0.31], *p* < 0.01; I^2^ = 0%, 95% CI [0–66%], *p* = 0.71). Also a significant reduction in overjet between baseline and end was demonstrated for brass instrument players when compared to controls (−0.51 mm, 95% CI [−0.95,−0.08], *p* = 0.02), however with high heterogeneity (I^2^ = 73%, 95% CI [24–90%], *p* = 0.01) and a 95% prediction interval with a wide range in opposite directions including the null [−2.32, 1.29]. For single reed instruments players vs. controls, the meta-analysis showed no significant effect on overjet. However, considerable heterogeneity was observed (I^2^ = 62–78%). Because the effect of brass and single reed instruments was diverged, pooling these two groups into one single experimental group was not feasible. Table 3 summarizes the detailed data outcomes of the performed meta-analysis. Online appendix 2a–c presents the corresponding forest plots.

**Table 3:** Meta-analysis on overjet for single-reed, brass and control at baseline and end scores

Comparison	Moment	Included studies	Model	DiffM	Test for overall		Test for heterogeneity				Prediction interval	Forest plot
					95% CI	*P*-value	I^2^ value % (95% CI)	Tau^2^	Chi^2^	*P*-value		Online appendix
Single-reed vs. control	Baseline	[5, 16, 25]	Random	−0.05	[−1.11, 1.01]	0.93	78% (28–93%)	0.69	8.99	0.01*	[−12.64, 12.54]	2a
	End	[5, 16, 25]	Random	0.01	[−0.79, 0.80]	0.99	62% (0–89%)	0.31	5.32	0.07*	[−8.71, 8.72]	2b
	Difference between baseline and end	[5, 16, 25]^a^	Random	−0.10	[−0.70, 0.49]	0.73	67% (0–91%)	0.18	6.15	0.05*	[−6.78, 6.57]	2c
Brass vs. control	Baseline	[5, 16, 25]	Random	−0.08	[−0.51, 0.36]	0.73	0% (0–74%)	0	1.77	0.62	[−1.02, 0.87]	2a
	End	[5, 16, 25]	Random	−0.67	[−1.02, −0.31]	<0.01*	0% (0–66%)	0	1.37	0.71	[−1.45, 0.12]	2b
	Difference between baseline and end	[5, 16, 25]^a^	Random	−0.51	[−0.95, −0.08]	0.02*	73% (24–90%)	0.13	11.10	0.01*	[−2.32, 1.29]	2c

*DiffM* difference of means; *CI* confidence interval

* statistically significant difference

^a^ Calculated by the authors of this review based on the data presented in the selected paper. To calculated the standard deviation of the difference score between baseline and end for the study of Brattström et al. [5] and Herman [16], a correlation of 0.5 was used

### Heterogeneity and sensitivity analysis

In the meta-analysis on end scores and difference scores of single-reed instrument players vs. controls, the studies were spread across both sides of the forest plot. This can be explained by the fact that large difference existed between the studies at baseline. For two studies [16, 25], the confidence interval at baseline did not contain 0, which implies a significant difference, however in opposite directions.

For the sensitivity analysis, outliers were explored as being the source of heterogeneity. In the meta-analysis for end scores of single-reed instrument players versus controls, the study of Pang [25] was an outlier. After excluding this study from the analysis, the heterogeneity was reduced to 0%. However, with the exclusion of this study, the baseline and difference scores remained heterogeneous (I^2^ = 78–83%). Therefore, it was concluded that the data for single reed instrument players vs. controls were inconsistent.

The meta-analysis for difference scores between baseline and end demonstrated significant more reduction in overjet for brass instrument players when compared to controls (−0.51 mm, 95% CI [−0.95,−0.08], *p* = 0.02), however with high heterogeneity (I^2^ = 73%, 95% CI [24–90%], *p* = 0.01). This heterogeneity could be reduced by excluding one outlier. The study by Brattström et al. analyzed boys and girls separately. For the sensitivity analysis, only the boys were included. This resulted in an effect size of −0.72 mm (95% CI [−1.00,−0.44], *p* < 0.01) and a heterogeneity of 0% (95% CI [0–11%]; *p* = 0.89) (Table 4). The overall result and conclusions were not affected by the sensitivity analyses although it did have an effect on the statistical heterogeneity expressed by I^2^. Consequently, the result of this review can be regarded with a higher degree of certainty. However the 95% prediction interval showed a wide range in opposite directions including the null [−2.56, 1.16].

**Table 4:** Sensitivity analysis on overjet for brass and control at baseline and end scores

Comparison	Moment	Included studies	Model	DiffM	Test for overall		Test for heterogeneity				Prediction interval	Forest plot
					95% CI	*P* value	I^2^ value % (95%CI)	Tau^2^	Chi^2^	*P *value		Online appendix
Brass vs. control	Baseline	[5, 25]; [16]º	Random	0.01	[−0.49, 0.50]	0.97	0% (0–84%)	0	1.29	0.52	[−3.20, 3.21]	3a
	End	[5, 25]; [16]º	Random	−0.73	[−1.14, −0.33]	<0.01*	0% (0–77%)	0	0.92	0.63	[−3.36, 1.89]	3b
	Difference between baseline and end	[5, 25]; [16]º^,a^	Random	−0.72	[−1.00, −0.44]	<0.01*	0% (0–11%)	0	0.23	0.89	[−2.56, 1.13]	3c

*DiffM* difference of means; *CI* confidence interval

* statistically significant difference

º This study analyzed boys and girls separately. For this sensitivity analysis, only the boys are included.

^a^ Calculated by the authors of this review based on the data presented in the selected paper. To calculated the standard deviation of the difference score between baseline and end for the study of Brattström et al. [5] and Herman [16], a correlation of 0.5 was used

### Synthesis of results

#### Tooth position

Overjet, overbite, incisor position and irregularity, posterior crossbite, intermolar width, and intercanine width showed (in some studies) significant differences between players of different types of wind instruments and controls (Fig. 3). With respect to other aspects, either no data was gathered or no significant differences were observed.

The data concerning overjet and overbite were inconsistent. Half of the studies showed no significant difference between wind players and controls. The outcomes of the studies that did observe an association indicated that adults who play a single-reed instrument had a larger overjet as compared to controls [14]. Three longitudinal studies [5, 16, 25] showed a reduction in overjet over time among children who played a brass instrument. Two of these longitudinal studies found a larger reduction in overjet in brass players as compared to controls [5, 25]. Data from these three longitudinal studies combined in a meta-analyses revealed that after a follow-up of 6 months to 3 years, brass instruments players had 0.72 mm more reduction in overjet when compared to controls (Table 3). One longitudinal study [16] found a larger reduction in overjet in flute players as compared to controls [16].

Cross-sectional data have shown that adults who play a wind instrument, when viewed as a group, have a smaller overbite as compared to controls [30]. However longitudinal data among children indicated that with most types of wind instruments some increase in overbite can be observed over time [16].

The reduction in overjet among children who play a brass instrument [5, 16, 25] does not seem to be caused by retroclination of the upper incisors, as longitudinal data showed that brass players developed more proclined upper incisors between the age of 6 and 15 years old as compared to controls [5]. Cross-sectional data showed that in adults and children who play a small brass instrument the upper incisor were more anteriorly displaced as compared to controls [30, 32], while in children who play a large brass instrument the lower incisors were more anteriorly displaced [32]. In children who play a single-reed instrument both upper and lower incisors were more anteriorly displaced as compared to controls [32]. In one cross-sectional study, adults who play a brass instrument showed a higher maxillary Little’s Irregularity Index score as compared to controls [1].

Although findings were not fully consistent, cross-sectional data showed that children who play a wind instrument had a higher prevalence of lingual crossbite of maxillary molars [6]. Maxillary intercanine width was found to be smaller among adults who play a wind instrument as compared to controls [30]. Also mandibular and maxillary intermolar width were found to be smaller among reed and flute players combined, as compared to controls [30]. Longitudinal data among children, however, showed that between the age of 6 and 9 in both single-reed and brass players, maxillary and mandibular intermolar width increased as compared to controls. At the age of 15 brass players had a wider mandibular and maxillary arch as compared to controls, while single-reed players only had a wider maxillary arch [5]. Cross-sectional data among adults showed that players of a large brass instrument had a lower ratio of maxillary intermolar width to mandibular intermolar width and a higher prevalence of lingual crossbite of the maxillary molars as compared to controls [13].

#### Facial morphology

**Fig. 5:**
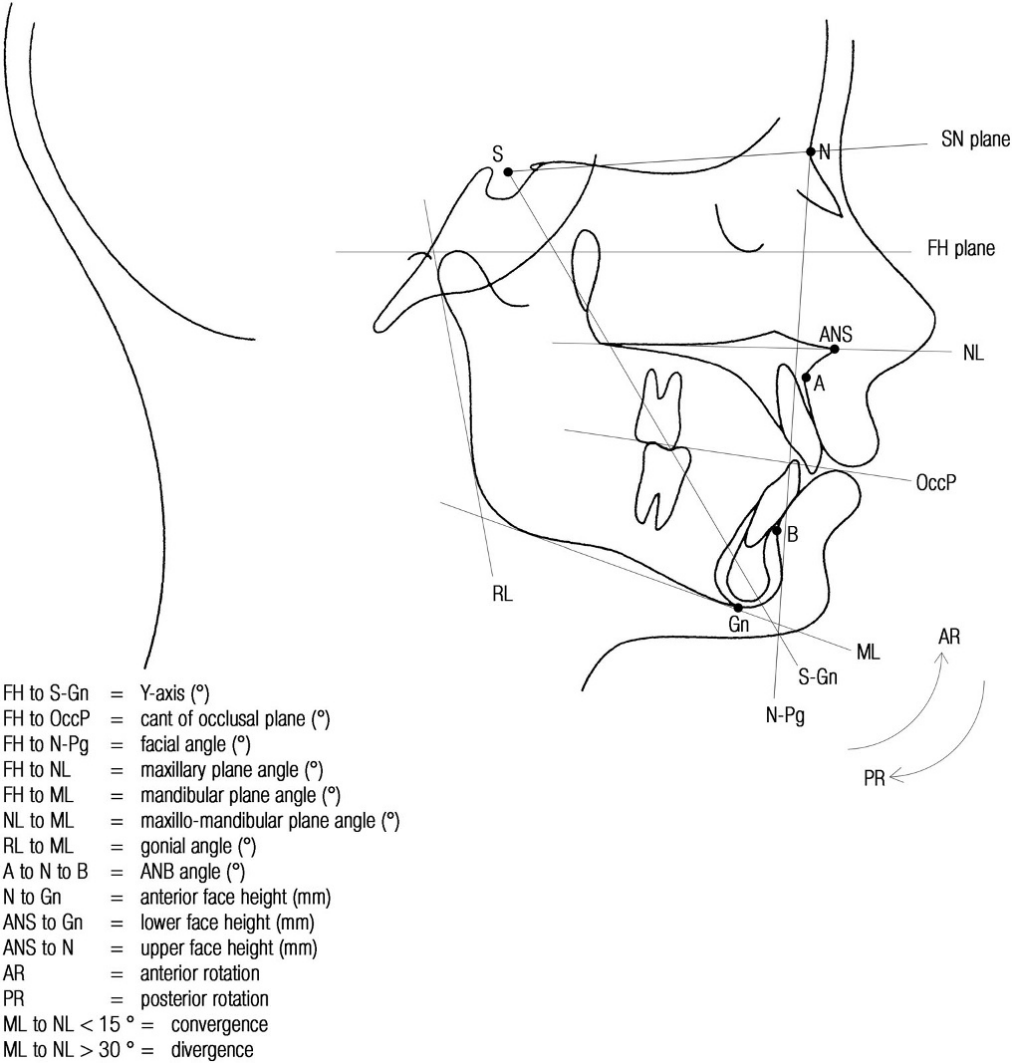
Cephalometric terminology

Fuhrimann et al. [10] found no significant difference in morphology of the face and the lips between adults who play the trumpet or clarinet and controls. However according to some studies, facial convergence/divergence (Fig. 5) and soft tissue thickness were aspects that showed significant differences between players of different types of wind instruments and controls (Fig. 4).

The data with respect to facial convergence/divergence were inconsistent. In a longitudinal study among Norwegian children, both brass and single-reed players showed less increase in anterior facial height between the age of 6 and 15 as compared to controls [5]. While the facial diagrams of this longitudinal study [5] showed an anterior rotation of the maxilla and mandible, a cross-sectional study among 13- to 18-year-old Japanese children [32] showed that single-reed players had a larger mandibular plane and maxillary plane angle as compared to controls [32]. Both the longitudinal study [5] and the cross-sectional study [32] showed that children who play a (large) brass instrument had a more anterior position of the maxilla and mandible as compared to controls. However, while longitudinal data [2] showed a greater reduction in maxillomandibular angle and mandibular plane angle among brass players as compared to controls, the cross-sectional study [32] showed that players of a small brass instrument had a larger maxillomandibular angle, mandibular plane angle and gonial angle. A larger gonial angle among brass players as compared to controls was also found in a cross-sectional study among adults [30].

One cross-sectional study among children [32] examined soft tissue thickness of wind instrument players as compared to controls. Flute players and players of a large brass or single-reed showed thicker upper lips as compared to controls. The lower lip was thicker among players of all types of wind instruments except for those of double-reed instruments.

### Rating the certainty and quality of the evidence

Table 5 summarizes the various factors used to rate strength of the evidence according to criteria as proposed by the GRADE working group [12, 15]. Although most studies examined professional or advanced wind instrumentalists, the generalizability was limited because most studies did not include all four types of wind instrument players. The outcomes of the included studies were inconsistent and therefore the degree of certainty surrounding the effect was rather imprecise. Furthermore, the potential risk of bias was estimated to be low to serious. Reporting bias could not be assessed, but could also not be ruled out. The magnitude of the effect was of questionable clinical relevance. Therefore, the strength of the evidence emerging from this systematic review was estimated to be “very low”.

**Table 5:** Summary of the estimated evidence profile (Grading of Recommendations Assessment, Development and Evaluation, GRADE) [12, 15]

Determinants of quality	Overall
Study design	Observational
Number of studies	10
Estimated potential risk of bias	Low to serious
Consistency	Inconsistent
Directness	Limited generalizability
Precision	Rather imprecise
Reporting bias	Cannot be ruled out
Magnitude of the effect	Of questionable clinical relevance
Strength of the evidence emerging from this review	Very low
Overall recommendation	Take into account a potential effect on overjet, arch width, facial divergence/convergence or lip thickness if an orthodontic patient plays a wind instrument

## Discussion

### Answer to the focused question

This is the first systematic review that evaluates the influence of playing a wind instrument on tooth position and facial morphology. Based on the data emerging from the studies included in this systematic review, the descriptive analysis showed that playing a wind instrument may have an effect on overjet, arch width, facial convergence/divergence, and soft tissue thickness. More specifically, playing a brass instrument was associated with a reduction in overjet among children, which could be substantiated in a meta-analysis. Furthermore, playing a brass instrument was associated with an increase in maxillary and mandibular intermolar width among children. Adults who play a large brass instrument had a higher prevalence of lingual crossbite of the maxillary molars. Children who play a wind instrument showed thicker lips as compared to controls.

Longitudinal data showed less increase in anterior facial height among both brass and single-reed players between the age of 6 and 15 years as compared to controls, as a result of an anterior rotation of the maxilla and mandible. This might depend on sample characteristics (age and ethnical background), as cross-sectional data among 13–18 year olds showed a tendency for posterior rotation of the maxilla and mandible or mandible alone for single-reed and brass players, respectively. However, to assess growth, a longitudinal study design is more appropriate which increases the reliability of the outcomes indicating an anterior rotation of the maxilla and mandible. However, to assess growth, the longitudinal study design is the most appropriate, which increases the reliability of the outcomes indicating that an anterior rotation of the maxilla and mandible can occur.

### Wind instruments as an aid in the treatment of malocclusions

According to Engelman [9], the pressure (lingually directed force of the upper lip against the maxillary incisors) exerted by the playing of reed instruments and flutes is higher than that by whistling, but lower than that by thumb-sucking and maximum muscle effort (the participant wind instrument player in Engelman’s study was instructed to pull the lip down as far as possible). On the other hand, the pressure exerted by playing brass instruments is higher than thumb-sucking and maximum muscle effort [9]. The first paper to theoretically propose that wind instruments may aid in the treatment of malocclusions suggested that *brass instruments* are indicated in cases of presenting hypotonicity of facial musculature and flabby lips, which can be found in Class II‑1 cases and Class I cases with protruding upper incisors [29]. Playing these instruments can exert a horizontal force on the maxillary and mandibular incisors that might result in retroclination of maxillary and mandibular incisors and lead to a reduction in overjet and an increase in overbite [13]. The selected studies indicated that indeed playing a brass instrument tended to reduce overjet, as compared to controls [5, 25]. However, none of the included studies showed that playing a brass instrument increases overbite. Instead of a retroclination of maxillary and mandibular incisors, three studies [5, 30, 32] showed that brass players had more proclined/anteriorly displaced upper and lower incisors compared to controls.

Strayer [34] also suggested that *single-reed instruments* would be indicated in Class III cases and contraindicated in Class I cases with protruding upper anterior teeth and in Class II cases. Playing a single-reed instrument can exert horizontal and vertical forces on the maxillary and mandibular incisors that might result in maxillary incisor proclination, mandibular incisor retroclination, and intrusion of maxillary and mandibular incisors, and therefore an increase in overjet and a reduction in overbite [13]. From the selected studies, it appeared that single-reed players may have larger overjets as compared to controls [14] and more anteriorly displaced maxillary incisors [32], although the meta-analysis showed inconsistency about the effect of playing a single reed instrument on overjet. Instead of a retroclination of mandibular incisors, one selected study [32] showed that single-reed players had more anteriorly displaced mandibular incisors. Evidence from the included studies did not suggest that playing a single-reed instrument would reduce overbite.

With respect to *double-reed instruments*, Strayer [34] suggested that playing such an instrument is indicated in all cases with hypotonic or short lips. Playing these instruments can exert horizontal and vertical forces on the maxillary and mandibular incisors that might result in retroclination and intrusion of maxillary and mandibular incisors, and therefore a reduction in overjet and overbite [13]. In contrast, one included study found that players of a double-reed instrument showed a significant increase in overbite after 2 years of playing [16] and had a higher prevalence of proclined maxillary incisors as compared to controls [32]. However, this was based on a sample size of only 4 double-reed players. None of the selected studies found that playing a double-reed instrument tended to increase overjet.

According to Strayer [34], the *flute* would be beneficial in Class I and Class III cases with short upper lips and unruly mentalis action, but contraindicated in Class II cases. The flute can exert a horizontal force on the mandibular incisors that might result in retroclination of mandibular incisors and therefore an increase in overjet [13]. In contrast, one included study found that flute players showed a significant decrease in overjet after 2 years of playing, which was significantly different from the control group [16].

### Confounders

Tooth movement requires application of forces exceeding a minimum threshold of magnitude and duration [13]. The duration of the force applied by wind instruments is an important factor to be considered. The number of years playing a wind instrument was assessed by 7 of the included studies [1, 5, 6, 13, 14, 16, 32] and 7 studies assessed the frequency of playing (hours per day) [1, 5, 6, 13, 14, 16, 32]. However, only one of them [1] evaluated whether there was an association. In that study, none of the outcomes were either influenced by the number of years or the frequency of play [1].

Professional musicians often play several different wind instruments. In these cases, the influence on the tooth position and facial morphology may be in different directions. This confounder was excluded by 3 of the included studies [5, 6, 13]. However, one of these studies included single-reed players who also played the flute but did not evaluate the impact of this confounder [13].

When wind instrumentalists have undergone or are currently undergoing orthodontic treatment, the influence of playing a wind instrument on tooth position and facial morphology cannot be estimated because it might attenuate the effects of playing a wind instrument on the dental arches. This confounder was excluded by 7 of the included studies [1, 5, 6, 13, 14, 16, 25].

Other confounders that were assessed or excluded by some of the included studies were the following: oral habits [1], digit-sucking habit [13, 14], tongue-thrust swallowing [14], mouth breathing [14], extractions of permanent teeth [1, 6, 13], dental restorations, periodontal disease, previous fractures of maxilla/mandible [1, 13], retained deciduous teeth or supernumerary teeth, dental cysts, pipe smoking [13], surgery to the temporomandibular joint (TMJ) [1], and adenoidectomy and/or tonsillectomy [14].

Gender, height, weight and Angle Class were the only potential confounders adjusted for in some of the studies. One study [25] reported that the participants were matched as to gender, height and weight. Because there were no male instrument players in the double-reed and flute group, the authors abstained to make comparisons with other groups. One study [5] analyzed data from boys and girls separately. Another study [32] did so too, but only because the girls were a small minority, and only for half of the data. One study [13] performed statistics to ascertain that gender was not a confounding factor for the significant differences found. It is very interesting that one study [16] analyzed the data from Class I cases and Class II cases separately and found that the reduction in overjet after 2 years of brass instrument playing was 0 mm for Class I cases and a significant −1.4 mm for Class II cases.

### Limitations of the individual included studies

For the individual included papers there were several limitations. Fuhrimann et al. [10] examined the morphology of the face, the dentition and the lips from profile cephalograms and dental casts. The authors reported that the same variables were measured as in a former article [36] of the last two authors, but did not report the outcomes for these variables. They only reported that they found no differences between trumpet or clarinet players and the controls.

Gualtieri [14] only performed statistics on the percentage of overbite and millimeters of overjet. Simple percentage comparisons were made for other outcomes. Accordingly, Herman [16] analyzed within group changes instead of between groups and only performed statistics with respect to change in millimeters of overbite and overjet. Other outcomes were reported descriptively. Pang [25] did not perform a statistical analysis at all.

The participants in the study of Adeyemi et al. [1] consisted only of males. Except for the study of Pang [25], the male–female ratio was unevenly distributed in the included studies. Two studies [13, 16] did not report information on gender distribution.

Shimada [32] used a control group that was not specified. Bwire [6] and Grammatopoulos et al. [13] had string players as part of the control group. This might have influenced the statistical difference between wind players and control group as a study performed by Kovero et al. [21] found that violin players had a larger face height (especially on the right side of the lower face and in the right mandibular ramus) and more proclined upper and lower incisors than controls. Two studies [14, 30] used dental care professionals/students as control group, which may have introduced a selection bias.

### Generalizability

As described in the introduction, the best approach is to distinguish four separate groups of wind instruments: brass, single-reed, double-reed, and flute (Fig. 1). Accordingly, this was done by four of the included studies [14, 16, 25, 32]. Unfortunately, in the study of Herman [16] and Shimada [32], only four double-reed players participated, and in the study of Pang [25], only two double-reed and four flute players were included. Four studies did not examine double-reed and flute players at all [1, 5, 10, 13]. One study did not examine double-reed players [6]. Rindisbacher et al. [30] regarded single-reed, double-reed, and flute as one group. Considering the various forms of embouchure, it is important for future research to investigate the different types of wind instruments separately with a sufficient number of players for each instrument type.

### Clinical relevance

Although the included studies showed significant differences between wind instrument players and controls, the magnitudes of these differences were small in some cases, making the clinical relevance questionable. For example in the study of Shimada [32], the maxillary plane angle was found to be 2° larger among single-reed instrument players as compared to controls. In other cases, the significant differences had clinical relevant magnitude, but showed a large variation. For example in the study of Shimada [32], the mandibular plane angle was found to be on average 6.8° larger among single-reed instrument players as compared to controls, however, with a standard deviation of 7.6°. Furthermore, a deviating jaw relationship will not necessarily have consequences for the dental and lip relationship because dentoalveolar compensation is possible. Similarly, the relevance of changes in arch width is questionable as long as the upper and lower arch fit together. The most clinically relevant variables might be overjet and overbite. For overbite there appeared to be no clear consensus between the included studies. Meta-analysis showed that after a follow-up of 6 months to 3 years, children who play a brass instrument had a significant reduction in overjet as compared to controls (−0.72 mm), but it remains questionable whether the magnitude of this difference was clinically relevant. In addition, the 95% prediction interval suggested that the effect could be null and with a wide range in opposite directions.

### Limitations of this review

A limitation of this systematic review is that only three longitudinal studies could be included. To assess the influence of playing a wind instrument on tooth position and facial morphology, this type of study is generally more appropriate than a cross-sectional study, to demonstrate a causal relationship. Cross sectional studies run the risk that the difference in outcome (or lack thereof) between exposed and unexposed can be explained entirely or partly by imbalance of other confounding causes.

Although no language filter was used, the possibility exists that some non-English papers were not added to the databases used. Seven potentially interesting non-English paper titles were identified by the search, of which the abstract and/or full text were not retrievable. One of these [24] found, as described in the abstract, that clarinetists tended to have large facial heights, which is in accordance with Brattström et al. [5], and tended to have lingually inclined lower incisors, which is not in agreement with Gualtieri [14]. The finding that clarinetists tended to have small mandibular intermolar widths [24] is in accordance with Rindisbacher et al. [30], but in contrast to Brattström et al. [5]. One paper was excluded after full text reading because tooth position was not examined clinically but via a questionnaire. In that study the most frequent dental change observed was (unspecified) inclination of the upper teeth [11]. Another paper was excluded because it lacked a control group of non-wind players [26]. This study found no difference between the average maxillary incisor angle of trumpet, clarinet, saxophone, and flute players.

While Strayer [34] recommended that specific wind instruments can be selected for their correcting influence on malocclusions, wind instruments can also be chosen to fit the prevailing malocclusion from the ease-of-playing standpoint [9]. If this was the case for the subjects in the included studies, it is a limitation in interpreting the outcomes of this systematic review. In line with this, the question arises as to what is the cause and what is the consequence: do clarinet players develop a large overjet by playing their instrument or do subjects with a larger overjet find the clarinet a more suitable instrument to play? For example, it has been reported that a wider dental arch had a significant positive relation with trumpet performance, whereas protrusion of upper incisors had a significant negative relation with trumpet performance [22, 37]. Interestingly this systematic review indicated that playing a brass instrument reduced overjet and widened the dental arch. A suggestion for future research is to inquire with the wind instrumentalists how they came about to choose their specific instrument of choice and whether their individual tooth position contributed to their choice.

The studies included in this systematic review involved either children [5, 16, 25, 32], adults [1, 10, 13, 14, 30] or both [6]. From the synthesis of results, it appeared that there might be a difference in the effect of playing a wind instrument on growing children and adults. To study this in more detail, future research is needed.

## Conclusion

Based on 10 observational studies, it can be concluded that playing a wind instrument can influence tooth position and facial morphology in both children and adults. Aspects that stand out are overjet, arch width, facial divergence/convergence, and lip thickness. However evidence was sparse. Due to a low to serious estimated potential risk of bias and a magnitude of questionable clinical relevance, the strength of the premises emerging from this review was graded to be “very low”. More longitudinal studies with parameters that allow for a meta-analysis would contribute to a better understanding of the impact of playing a wind instrument on tooth position and facial morphology.

## Caption Electronic Supplementary Material

Online Appendix 1. Risk of Bias assessment. Online Appendix 2. Forest plots of meta-analysis. Online Appendix 3. Forest plots of sensitivity analysis. Online Appendix 4. R markdown pdf document

Online appendix 4
