# Supplementary material for: Does playing a wind instrument influence tooth position and facial morphology? Systematic review and meta-analysis
Source: J Orofac Orthop. 2020 May 7;81(4):267–85. doi: 10.1007/s00056-020-00223-9 (PMC7316676; doi:10.1007/s00056-020-00223-9)

Online Appendix 1. Risk of Bias assessment using a comprehensive combination of criteria as suggested by the critical appraisal checklist for analytical cross-sectional studies [20], the Newcastle Ottawa scale adapted for cross sectional studies [18] and the ROBINS-I tool [4]. Judgement of risk of bias is presented according to the 7 domains as suggested by the ROBINS-I tool.

**Included studies:**	[1]	[5]	[6]	[7]	[13]	[14]	[16]	[25]	[30]	[33]
**<u>Pre-assessment domains</u>**										
**1. Bias due to confounding**										
■ Were confounding factors defined?	Yes	Yes	Yes	Yes	Yes	Yes	Yes	Yes	Yes	Yes
■ Were confounding factors assessed?	Yes	Yes	Yes	Yes	Yes	Yes	Yes	Yes	Yes	Yes
■ Were strategies to deal with confounding factors stated?	Yes	No	No	No	Yes	No	No	Yes	No	No
Risk of bias judgement	*Low*	*Moderate*	*Moderate*	*Moderate*	*Low*	*Moderate*	*Moderate*	*Low*	*Moderate*	*Moderate*
**2. Bias in selection of participants into the study**										
■ Were the criteria for inclusion in the sample clearly defined?	Yes	Yes	Yes	Yes	Yes	Yes	Yes	Yes	Yes	Yes
■ Were the study subjects described in detail?	Yes	Yes	Yes	Yes	No	Yes	Yes	Yes	Yes	Yes
■ Is the study sample representative of the average in the target population?	No	No	No	No	No	Yes	Yes	No	No	Yes
■ Is the sample size justified and satisfactory?	Yes	No	No	No	Yes	No	No	No	No	No
Risk of bias judgement	*Moderate*	*Serious*	*Serious*	*Serious*	*Serious*	*Moderate*	*Moderate*	*Serious*	*Serious*	*Moderate*
**3. Bias in classification of condition (wind instrument playing)**										
Risk of bias judgement	*NA*	*NA*	*NA*	*NA*	*NA*	*NA*	*NA*	*NA*	*NA*	*NA*
**<u>Post-assessment domains</u>**										
**4. Bias due to deviations from intended intervention**										
Risk of bias judgement	*NA*	*NA*	*NA*	*NA*	*NA*	*NA*	*NA*	*NA*	*NA*	*NA*

**5. Bias due to missing data**										
■ Were outcome data available for (nearly) all participants?	Yes	Yes	Yes	Yes	Yes	Yes	No	Yes	Yes	Yes
Risk of bias judgement	*Low*	*Low*	*Low*	*Low*	*Low*	*Low*	*Critical*	*Low*	*Low*	*Low*
**6. Bias in measurement of outcomes (tooth position and facial morphology)**										
■ Were the investigators blinded to the condition?	Yes	NR	NR	NR	Yes	NR	NR	Yes	NR	NR
■ Were the investigators calibrated?	Yes	NR	Yes	NR	NR	NR	NR	NR	NR	NR
■Were the outcomes measured in a valid and reliable way?	Yes	Yes	Yes	Yes	Yes	NR	Yes	Yes	Yes	Yes
■ Was appropriate statistical analysis used?	Yes	Yes	Yes	Yes	Yes	No	No	No	Yes	Yes
Risk of bias judgement	*Low*	*Serious*	*Moderate*	*Serious*	*Moderate*	*Critical*	*Critical*	*Serious*	*Serious*	*Serious*
**7. Bias in selection of the reported result**										
■ Are the reported effect estimates based on the results?	Yes	Yes	Yes	Yes	Yes	Yes	Yes	Yes	Yes	Yes
Risk of bias judgement	*Low*	*Low*	*Low*	*Low*	*Low*	*Low*	*Low*	*Low*	*Low*	*Low*
**OVERALL Risk of bias**	**Low**	**Moderate**	**Moderate**	**Moderate**	**Moderate**	**Serious**	**Serious**	**Moderate**	**Moderate**	**Moderate**

Judgements: Low, moderate, serious, critical, not reported (NR), not applicable (NA)

[1] Systematic oral examination record form used in the Orthodontic Clinic at the University of Michigan

Online Appendix 2a. Forest plot of meta-analysis (random) on overjet for single-reed (SR) and brass (B) vs. control at baseline scores

Study or Subgroup	Windinstrument Mean	Windinstrument SD	Windinstrument Total	Control Mean	Control SD	Control Total	Weight	Mean Difference IV, Random, 95% CI
**Single reed**								
Brattstrom SR girls 1989	4.53	1.71	20	3.48	0.93	10	13.1%	1.05 [0.10; 2.00]
Herman SR 1981	2.50	1.63	41	2.76	1.34	18	15.9%	−0.26 [−1.06; 0.54]
Pang SR 1976	3.99	0.94	21	4.88	1.53	15	14.4%	−0.89 [−1.76; −0.02]
**Total (random effects, 95% CI)**			**82**			**43**	**43.4%**	**−0.05 [−1.11; 1.01]**
Heterogeneity: Tau^2 = 0.6867; Chi^2 = 8.99, df = 2 (P = 0.01); I^2 = 78% [28%; 93%]								
Test for overall effect: Z = −0.09 (P = 0.927)								
**Brass**								
Brattstrom B boys 1989	3.83	1.31	18	4.05	0.79	20	18.0%	−0.22 [−0.92; 0.48]
Brattstrom B girls 1989	3.13	1.54	20	3.48	0.93	10	14.1%	−0.35 [−1.24; 0.54]
Herman B 1981	2.80	1.57	21	2.76	1.34	18	13.6%	0.04 [−0.87; 0.95]
Pang B 1976	5.41	1.72	19	4.88	1.53	15	10.9%	0.53 [−0.56; 1.62]
**Total (random effects, 95% CI)**			**78**			**63**	**56.6%**	**−0.08 [−0.51; 0.36]**
Heterogeneity: Tau^2 = 0; Chi^2 = 1.77, df = 3 (P = 0.62); I^2 = 0% [0%; 74%]								
Test for overall effect: Z = −0.34 (P = 0.731)								
**Total (random effects, 95% CI)**			**160**			**106**	**100.0%**	**−0.06 [−0.50; 0.38]**
**Prediction interval**								**[−1.23; 1.11]**

Heterogeneity: Tau^2 = 0.1562; Chi^2 = 10.76, df = 6 (P = 0.10); I^2 = 44% [0%; 77%]

Residual heterogeneity: Tau^2 = NA; Chi^2 = 10.76, df = 5 (P = 0.06); I^2 = 54% [0%; 81%]

Test for overall effect: Z = −0.25 (P = 0.799)

Test for subgroup differences: Chi^2 = 0.00, df = 1 (P = 0.96)

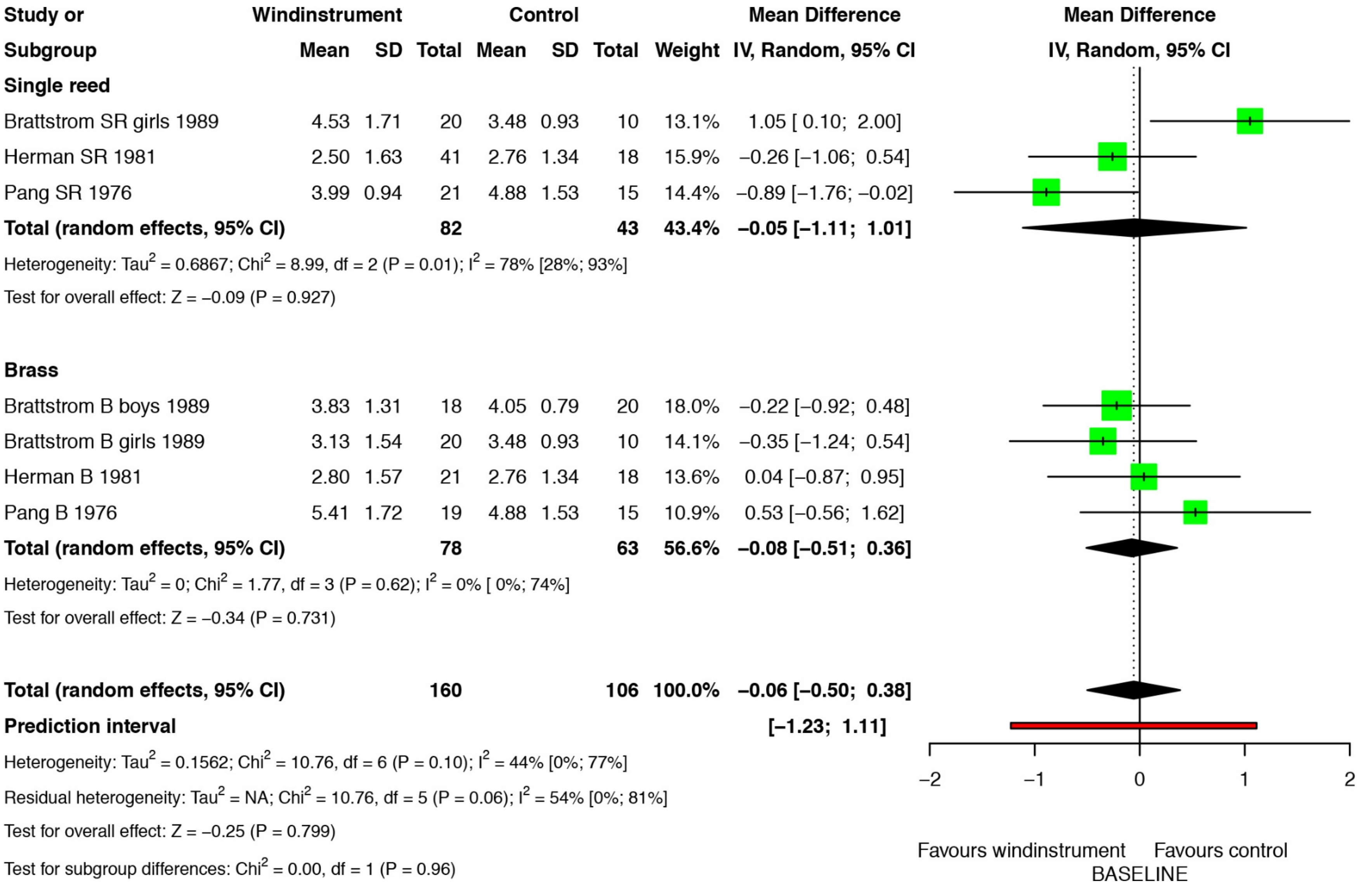

Online Appendix 2b. Forest plot of meta-analysis (random) on overjet for single-reed (SR) and brass (B) vs. control at end scores

Study or Subgroup	Windinstrument Mean	SD	Total	Control Mean	SD	Total	Weight	Mean Difference IV, Random, 95% CI
**Single reed**								
Brattstrom SR girls 1989	3.79	1.28	20	3.28	0.92	10	14.3%	0.51 [–0.29; 1.31]
Herman SR 1981	2.92	1.82	41	2.65	0.95	18	15.9%	0.27 [–0.44; 0.98]
Pang SR 1976	3.81	1.07	21	4.80	1.88	15	10.5%	–0.99 [–2.05; 0.07]
**Total (random effects, 95% CI)**			**82**			**43**	**40.7%**	**0.01 [–0.79; 0.80]**
Heterogeneity: $Tau^2 = 0.3059$; $Chi^2 = 5.32$, df = 2 (P = 0.07); $I^2 = 62\%$ [0%; 89%]								
Test for overall effect: Z = 0.01 (P = 0.988)								
**Brass**								
Brattstrom B boys 1989	3.19	0.99	18	4.11	0.81	20	18.5%	–0.92 [–1.50; –0.34]
Brattstrom B girls 1989	2.84	1.12	20	3.28	0.92	10	15.1%	–0.44 [–1.19; 0.31]
Herman B 1981	2.05	1.07	21	2.65	0.95	18	17.4%	–0.60 [–1.23; 0.03]
Pang B 1976	4.45	1.85	19	4.80	1.88	15	8.3%	–0.35 [–1.61; 0.91]
**Total (random effects, 95% CI)**			**78**			**63**	**59.3%**	**–0.67 [–1.02; –0.31]**
Heterogeneity: $Tau^2 = 0$; $Chi^2 = 1.37$, df = 3 (P = 0.71); $I^2 = 0\%$ [0%; 66%]								
Test for overall effect: Z = –3.66 (P < 0.001)								
**Total (random effects, 95% CI)**			**160**			**106**	**100.0%**	**–0.36 [–0.79; 0.08]**
**Prediction interval**								**[–1.59; 0.88]**
Heterogeneity: $Tau^2 = 0.1807$; $Chi^2 = 13.09$, df = 6 (P = 0.04); $I^2 = 54\%$ [0%; 80%]								
Residual heterogeneity: Tau^2 = NA; $Chi^2 = 6.69$, df = 5 (P = 0.24); $I^2 = 25\%$ [0%; 69%]								
Test for overall effect: Z = –1.61 (P = 0.107)								
Test for subgroup differences: $Chi^2 = 2.28$, df = 1 (P = 0.13)								

Mean Difference
IV, Random, 95% CI

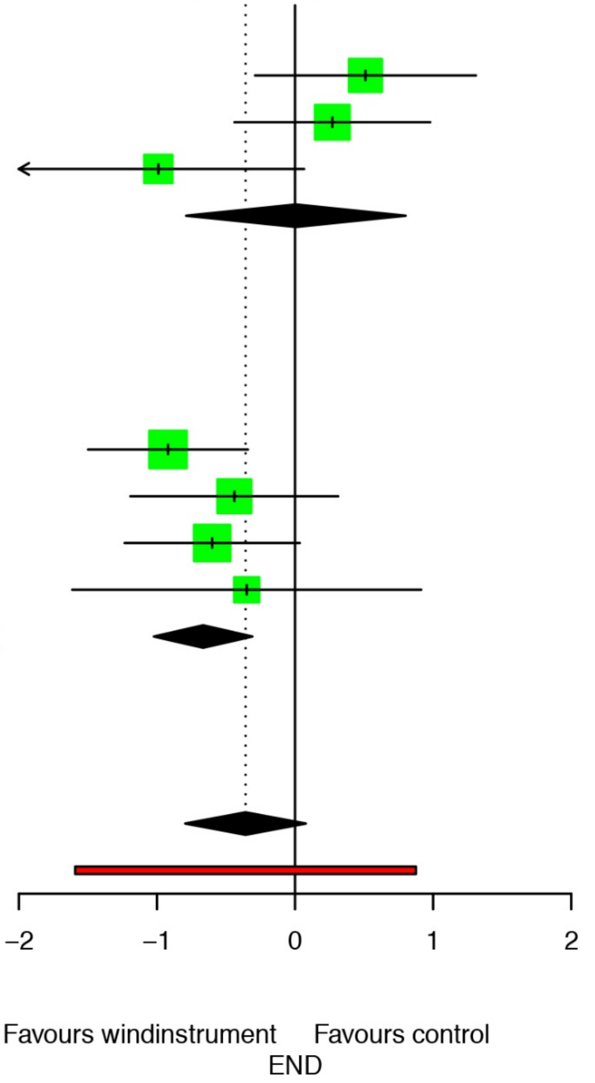

Online Appendix 2c. Forest plot of meta-analysis (random) on overjet for single-reed (SR) and brass (B) vs. control for difference scores between baseline and end

Study or Subgroup	Windinstrument Mean	SD	Total	Control Mean	SD	Total	Weight	Mean Difference IV, Random, 95% CI
**Single reed**								
Brattstrom SR girls 1989	–0.74	0.88	20	–0.20	0.23	10	17.2%	–0.54 [–0.95; –0.13]
Herman SR 1981	0.42	1.73	41	–0.11	1.19	18	10.0%	0.53 [–0.23; 1.29]
Pang SR 1976	–0.18	0.96	21	–0.08	0.92	15	12.5%	–0.10 [–0.72; 0.52]
**Total (random effects, 95% CI)**			**82**			**43**	**39.7%**	**–0.10 [–0.70; 0.49]**
Heterogeneity: Tau^2 = 0.1842; Chi^2 = 6.15, df = 2 (P = 0.05); I^2 = 67% [0%; 91%]								
Test for overall effect: Z = –0.35 (P = 0.730)								
**Brass**								
Brattstrom B boys 1989	–0.64	0.72	18	0.06	0.07	20	19.2%	–0.70 [–1.03; –0.37]
Brattstrom B girls 1989	–0.29	0.45	20	–0.20	0.23	10	21.4%	–0.09 [–0.33; 0.15]
Herman B 1981	–0.75	1.39	21	–0.11	1.19	18	9.3%	–0.64 [–1.45; 0.17]
Pang B 1976	–0.96	1.26	19	–0.08	0.92	15	10.5%	–0.88 [–1.61; –0.15]
**Total (random effects, 95% CI)**			**78**			**63**	**60.3%**	**–0.51 [–0.95; –0.08]**
Heterogeneity: Tau^2 = 0.1275; Chi^2 = 11.1, df = 3 (P = 0.01); I^2 = 73% [24%; 90%]								
Test for overall effect: Z = –2.33 (P = 0.020)								
**Total (random effects, 95% CI)**			**160**			**106**	**100.0%**	**–0.36 [–0.67; –0.04]**
**Prediction interval**								**[–1.28; 0.57]**
Heterogeneity: Tau^2 = 0.1042; Chi^2 = 17.58, df = 6 (P < 0.01); I^2 = 66% [24%; 85%]								
Residual heterogeneity: Tau^2 = NA; Chi^2 = 17.24, df = 5 (P < 0.01); I^2 = 71% [33%; 88%]								
Test for overall effect: Z = –2.24 (P = 0.025)								
Test for subgroup differences: Chi^2 = 1.19, df = 1 (P = 0.27)								

Mean Difference IV, Random, 95% CI

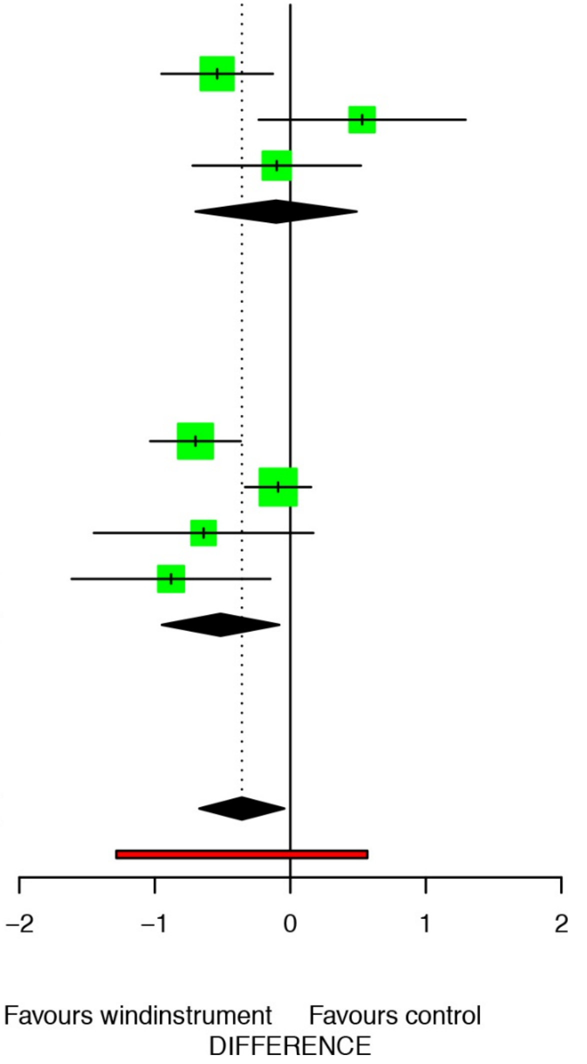

Online Appendix 3a. Forest plot of sensitivity analysis (random) on overjet for brass (B) vs. control at baseline scores

Study or Subgroup	Windinstrument Mean	SD	Total	Control Mean	SD	Total	Weight	Mean Difference IV, Random, 95% CI
**Brass**								
Brattstrom B boys 1989	3.83	1.31	18	4.05	0.79	20	20.3%	–0.22 [–0.92; 0.48]
Herman B 1981	2.80	1.57	21	2.76	1.34	18	16.0%	0.04 [–0.87; 0.95]
Pang B 1976	5.41	1.72	19	4.88	1.53	15	13.1%	0.53 [–0.56; 1.62]
**Total (random effects, 95% CI)**			**58**			**53**	**49.5%**	**0.01 [–0.49; 0.50]**

Heterogeneity: $Tau^2 = 0$; $Chi^2 = 1.29$, df = 2 (P = 0.52); $I^2 = 0\%$ [0%; 84%]

Test for overall effect: Z = 0.04 (P = 0.97)

Mean Difference IV, Random, 95% CI

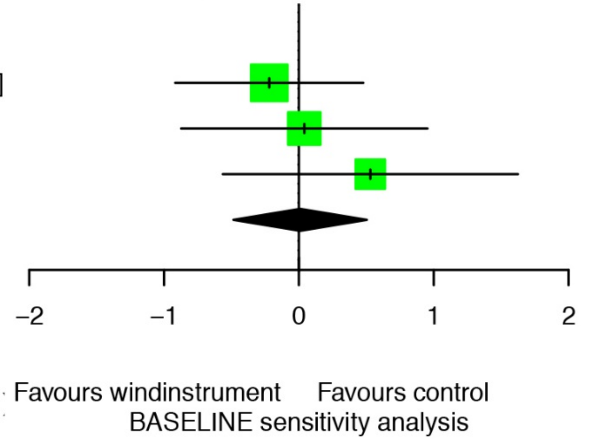

Online Appendix 3b. Forest plot of sensitivity analysis (random effects) on overjet for brass (B) vs. control at end scores

Study or Subgroup	Windinstrument Mean	SD	Total	Control Mean	SD	Total	Weight	Mean Difference IV, Random, 95% CI
**Brass**								
Brattstrom B boys 1989	3.19	0.99	18	4.11	0.81	20	20.9%	−0.92 [−1.50; −0.34]
Herman B 1981	2.05	1.07	21	2.65	0.95	18	19.9%	−0.60 [−1.23; 0.03]
Pang B 1976	4.45	1.85	19	4.80	1.88	15	10.6%	−0.35 [−1.61; 0.91]
**Total (random effects, 95% CI)**			**58**			**53**	**51.4%**	**−0.73 [−1.14; −0.33]**

Heterogeneity: $Tau^2 = 0$; $Chi^2 = 0.92$, df = 2 (P = 0.63); $I^2 = 0\%$ [0%; 77%]

Test for overall effect: Z = −3.54 (P < 0.01)

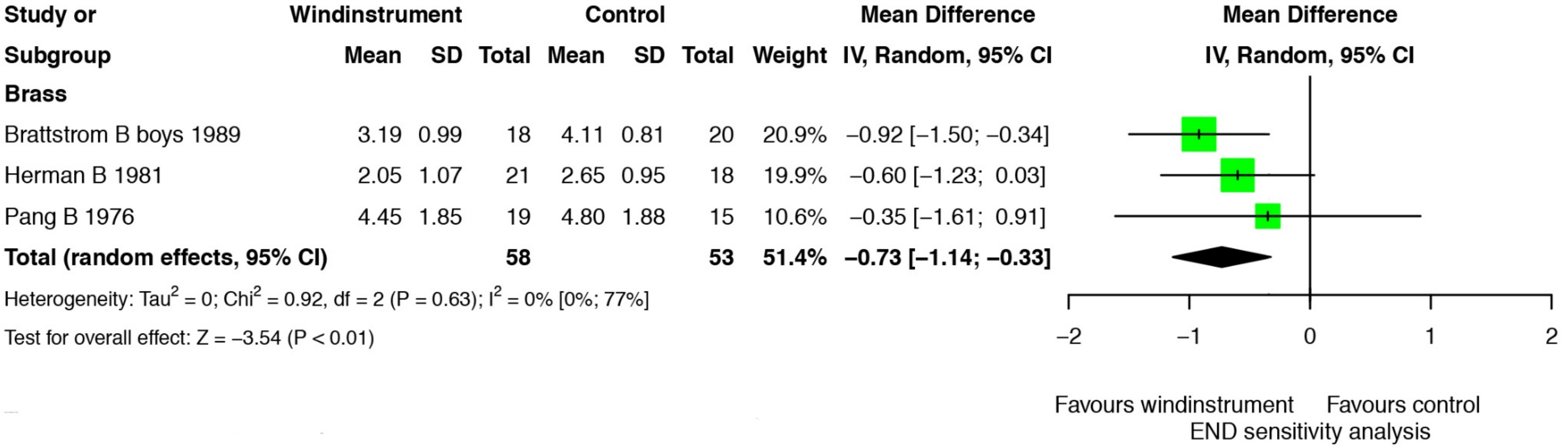

Online Appendix 3c. Forest plot of sensitivity analysis (random) on overjet for brass (B) vs. control for difference scores between baseline and end

Study or Subgroup	Windinstrument Mean	Windinstrument SD	Windinstrument Total	Control Mean	Control SD	Control Total	Weight	Mean Difference IV, Random, 95% CI
**Brass**								
Brattstrom B boys 1989	−0.64	0.72	18	0.06	0.07	20	24.8%	−0.70 [−1.03; −0.37]
Herman B 1981	−0.75	1.39	21	−0.11	1.19	18	11.6%	−0.64 [−1.45; 0.17]
Pang B 1976	−0.96	1.26	19	−0.08	0.92	15	13.1%	−0.88 [−1.61; −0.15]
**Total (random effects, 95% CI)**			**58**			**53**	**49.6%**	**−0.72 [−1.00; −0.44]**

Heterogeneity: $Tau^2 = 0$; $Chi^2 = 0.23$, df = 2 (P = 0.89); $I^2 = 0\%$ [0%; 11%]

Test for overall effect: Z = −4.96 (P < 0.01)

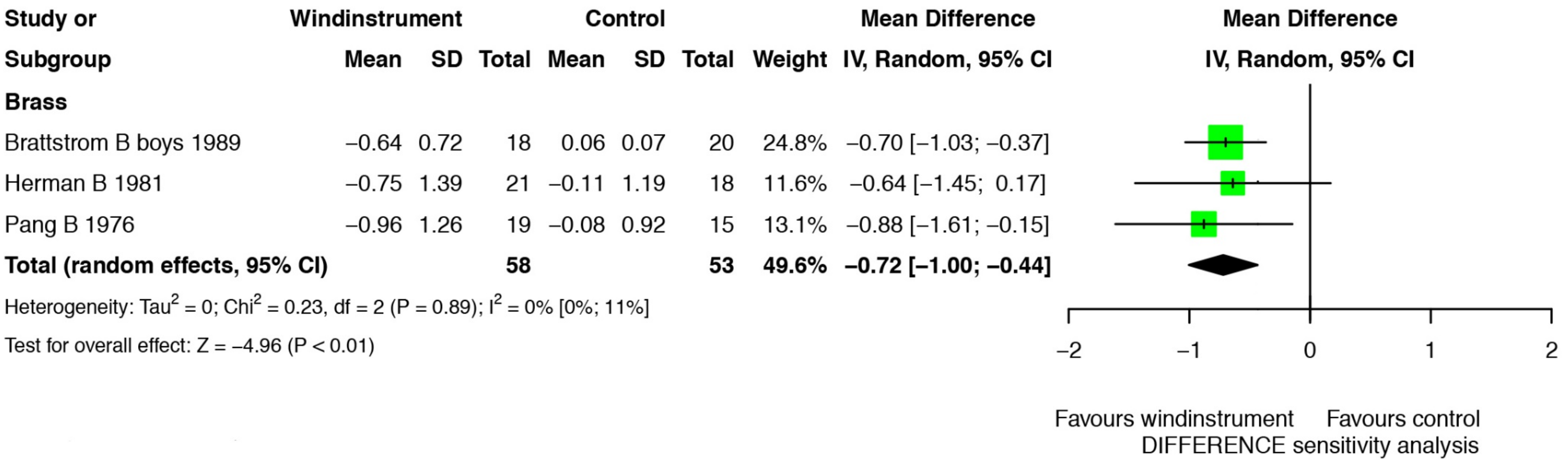

Supplement: Supplementary file 1 — Online Appendix 1. Risk of Bias assessment. Online Appendix 2. Forest plots of meta-analysis. Online Appendix 3. Forest plots of sensitivity analysis. Online Appendix 4. R markdown pdf document [file 56_2020_223_MOESM1_ESM.pdf]
