# Supplementary material for: Does playing a wind instrument influence tooth position and facial morphology? Systematic review and meta-analysis
Source: J Orofac Orthop. 2020 May 7;81(4):267–85. doi: 10.1007/s00056-020-00223-9 (PMC7316676; doi:10.1007/s00056-020-00223-9)
Supplement: Supplementary file 2 — Online appendix 4 [file 56_2020_223_MOESM2_ESM.pdf]

ACADEMISCH CENTRUM TANDHEELKUNDE AMSTERDAM

SR Fawn van der Weijden

R Statistics Cees Valkenburg

06-09-2019

Contents

BASELINE **2**
Data BASELINE . . . 2
Plot BASELINE with subgroups random effects . . . 3
BASELINE analysis subgroup Single Reed . . . 4
BASELINE analysis subgroup Brass . . . 5
Plot BASELINE sensitivity analysis (without Brattstrom B girls) . . . 6
BASELINE sensitivity analysis subgroup Brass . . . 7

END **9**
Data END . . . 9
Plot END with subgroups random effects . . . 10
END analysis subgroup Single Reed . . . 11
END analysis subgroup Brass . . . 12
Plot END sensitivity analysis (without Brattstrom B girls) . . . 13
END sensitivity analysis subgroup Brass . . . 14

DIFFERENCE **16**
Data DIFFERENCE . . . 16
Plot DIFFERENCE with subgroups random effects . . . 17
DIFFERENCE analysis subgroup Single Reed . . . 18
DIFFERENCE analysis subgroup Brass . . . 19
Plot DIFFERENCE sensitivity analysis (without Brattstrom B girls) . . . 20
DIFFERENCE sensitivity analysis subgroup Brass . . . 21

References **23**

- —

BASELINE

Data BASELINE

```
## # A tibble: 7 x 10
##    ...1 author            year    Ne    Me    Se    Nc    Mc    Sc sub
##   <dbl> <chr>            <dbl> <dbl> <dbl> <dbl> <dbl> <dbl> <dbl> <chr>
## 1     1 Brattstrom SR ~  1989    20  4.53  1.71    10  3.48  0.93 Single r~
## 2     2 Herman SR        1981    41  2.5   1.63    18  2.76  1.34 Single r~
## 3     3 Pang SR          1976    21  3.99  0.94    15  4.88  1.53 Single r~
## 4     4 Brattstrom B b~  1989    18  3.83  1.31    20  4.05  0.79 Brass
## 5     5 Brattstrom B g~  1989    20  3.13  1.54    10  3.48  0.93 Brass
## 6     6 Herman B         1981    21  2.8   1.57    18  2.76  1.34 Brass
## 7     7 Pang B           1976    19  5.41  1.72    15  4.88  1.53 Brass
```

Plot BASELINE with subgroups random effects

Study or Subgroup	Windinstrument Mean	SD	Total	Control Mean	SD	Total	Weight	Mean Difference IV, Random, 95% CI
**Single reed**								
Brattstrom SR girls 1989	4.53	1.71	20	3.48	0.93	10	13.1%	1.05 [0.10; 2.00]
Herman SR 1981	2.50	1.63	41	2.76	1.34	18	15.9%	–0.26 [–1.06; 0.54]
Pang SR 1976	3.99	0.94	21	4.88	1.53	15	14.4%	–0.89 [–1.76; –0.02]
**Total (random effects, 95% CI)**			**82**			**43**	**43.4%**	**–0.05 [–1.11; 1.01]**
Heterogeneity: Tau^2 = 0.6867; Chi^2 = 8.99, df = 2 (P = 0.01); I^2 = 78% [28%; 93%]								
Test for overall effect: Z = –0.09 (P = 0.927)								
**Brass**								
Brattstrom B boys 1989	3.83	1.31	18	4.05	0.79	20	18.0%	–0.22 [–0.92; 0.48]
Brattstrom B girls 1989	3.13	1.54	20	3.48	0.93	10	14.1%	–0.35 [–1.24; 0.54]
Herman B 1981	2.80	1.57	21	2.76	1.34	18	13.6%	0.04 [–0.87; 0.95]
Pang B 1976	5.41	1.72	19	4.88	1.53	15	10.9%	0.53 [–0.56; 1.62]
**Total (random effects, 95% CI)**			**78**			**63**	**56.6%**	**–0.08 [–0.51; 0.36]**
Heterogeneity: Tau^2 = 0; Chi^2 = 1.77, df = 3 (P = 0.62); I^2 = 0% [0%; 74%]								
Test for overall effect: Z = –0.34 (P = 0.731)								
**Total (random effects, 95% CI)**			**160**			**106**	**100.0%**	**–0.06 [–0.50; 0.38]**
**Prediction interval**								**[–1.23; 1.11]**

Heterogeneity: Tau^2 = 0.1562; Chi^2 = 10.76, df = 6 (P = 0.10); I^2 = 44% [0%; 77%]

Residual heterogeneity: Tau^2 = NA; Chi^2 = 10.76, df = 5 (P = 0.06); I^2 = 54% [0%; 81%]

Test for overall effect: Z = –0.25 (P = 0.799)

Test for subgroup differences: Chi^2 = 0.00, df = 1 (P = 0.96)

Mean Difference
IV, Random, 95% CI

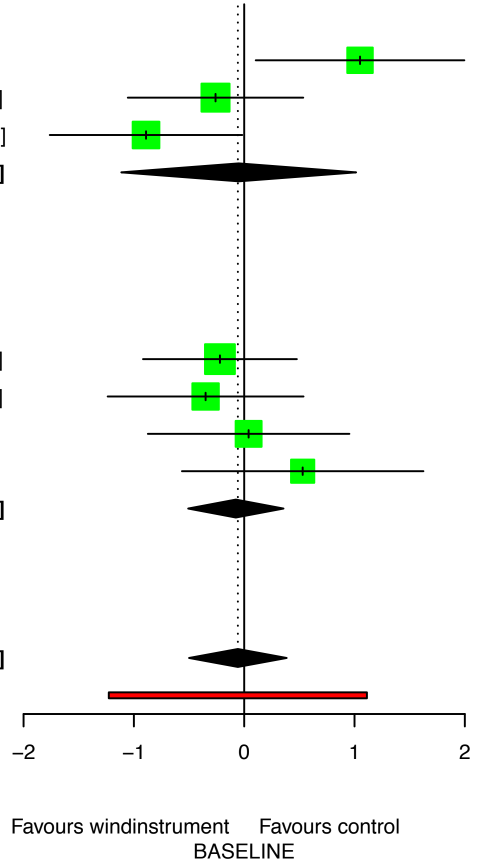

BASELINE analysis subgroup Single Reed

```
##                              MD          95%-CI %W(fixed) %W(random)
## Brattstrom SR girls 1989  1.05 [ 0.10;  2.00]      27.9       32.1
## Herman SR 1981           -0.26 [-1.06;  0.54]      39.4       34.6
## Pang SR 1976             -0.89 [-1.76; -0.02]      32.7       33.3
##
## Number of studies combined: k = 3
##
##                          MD            95%-CI     z p-value
## Fixed effect model   -0.10 [ -0.60;  0.40] -0.40  0.6913
## Random effects model -0.05 [ -1.11;  1.01] -0.09  0.9268
## Prediction interval        [-12.64; 12.54]
##
## Quantifying heterogeneity:
## tau^2 = 0.6867; H = 2.12 [1.18; 3.81]; I^2 = 77.8% [28.0%; 93.1%]
##
## Test of heterogeneity:
##     Q d.f. p-value
##  8.99    2  0.0112
##
## Details on meta-analytical method:
## - Inverse variance method
## - DerSimonian-Laird estimator for tau^2
```

BASELINE analysis subgroup Brass

```
##                             MD         95%-CI %W(fixed) %W(random)
## Brattstrom B boys 1989  -0.22 [-0.92; 0.48]      38.4       38.4
## Brattstrom B girls 1989 -0.35 [-1.24; 0.54]      23.7       23.7
## Herman B 1981            0.04 [-0.87; 0.95]      22.4       22.4
## Pang B 1976              0.53 [-0.56; 1.62]      15.6       15.6
##
## Number of studies combined: k = 4
##
##                          MD         95%-CI     z p-value
## Fixed effect model    -0.08 [-0.51; 0.36] -0.34  0.7308
## Random effects model -0.08 [-0.51; 0.36] -0.34  0.7308
## Prediction interval         [-1.02; 0.87]
##
## Quantifying heterogeneity:
## tau^2 = 0; H = 1.00 [1.00; 1.96]; I^2 = 0.0% [0.0%; 74.0%]
##
## Test of heterogeneity:
##     Q d.f. p-value
##  1.77    3  0.6215
##
## Details on meta-analytical method:
## - Inverse variance method
## - DerSimonian-Laird estimator for tau^2
```

Plot BASELINE sensitivity analysis (without Brattstrom B girls)

Study or Subgroup	Windinstrument Mean	SD	Total	Control Mean	SD	Total	Weight	Mean Difference IV, Random, 95% CI
**Single reed**								
Brattstrom SR girls 1989	4.53	1.71	20	3.48	0.93	10	15.5%	1.05 [0.10; 2.00]
Herman SR 1981	2.50	1.63	41	2.76	1.34	18	18.3%	−0.26 [−1.06; 0.54]
Pang SR 1976	3.99	0.94	21	4.88	1.53	15	16.8%	−0.89 [−1.76; −0.02]
**Total (random effects, 95% CI)**			**82**			**43**	**50.5%**	**−0.05 [−1.11; 1.01]**
Heterogeneity: $Tau^2 = 0.6867$; $Chi^2 = 8.99$, df = 2 (P = 0.01); $I^2 = 78\%$ [28%; 93%]								
Test for overall effect: Z = −0.09 (P = 0.93)								
**Brass**								
Brattstrom B boys 1989	3.83	1.31	18	4.05	0.79	20	20.3%	−0.22 [−0.92; 0.48]
Herman B 1981	2.80	1.57	21	2.76	1.34	18	16.0%	0.04 [−0.87; 0.95]
Pang B 1976	5.41	1.72	19	4.88	1.53	15	13.1%	0.53 [−0.56; 1.62]
**Total (random effects, 95% CI)**			**58**			**53**	**49.5%**	**0.01 [−0.49; 0.50]**
Heterogeneity: $Tau^2 = 0$; $Chi^2 = 1.29$, df = 2 (P = 0.52); $I^2 = 0\%$ [0%; 84%]								
Test for overall effect: Z = 0.04 (P = 0.97)								
**Total (random effects, 95% CI)**			**140**			**96**	**100.0%**	**−0.00 [−0.52; 0.51]**
**Prediction interval**								**[−1.47; 1.46]**
Heterogeneity: $Tau^2 = 0.2105$; $Chi^2 = 10.37$, df = 5 (P = 0.07); $I^2 = 52\%$ [0%; 81%]								
Test for overall effect: Z = −0.01 (P = 0.99)								
Test for subgroup differences: $Chi^2 = 0.01$, df = 1 (P = 0.92)								

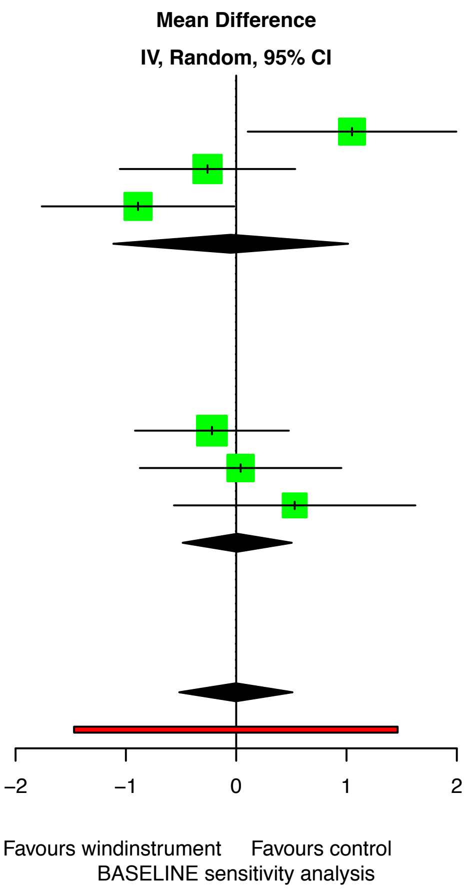

BASELINE sensitivity analysis subgroup Brass

```
##                            MD        95%-CI %W(fixed) %W(random)   sub
## Brattstrom B boys 1989 -0.22 [-0.92; 0.48]      50.3       50.3 Brass
## Herman B 1981           0.04 [-0.87; 0.95]      29.3       29.3 Brass
## Pang B 1976             0.53 [-0.56; 1.62]      20.4       20.4 Brass
##
## Number of studies combined: k = 3
##
##                         MD        95%-CI    z p-value
## Fixed effect model    0.01 [-0.49; 0.50] 0.04  0.9707
## Random effects model 0.01 [-0.49; 0.50] 0.04  0.9707
## Prediction interval        [-3.20; 3.21]
##
## Quantifying heterogeneity:
## tau^2 = 0; H = 1.00 [1.00; 2.49]; I^2 = 0.0% [0.0%; 83.9%]
##
## Quantifying residual heterogeneity:
## H = 1.00 [1.00; 2.49]; I^2 = 0.0% [0.0%; 83.9%]
##
## Test of heterogeneity:
##     Q d.f. p-value
##  1.29    2  0.5248
##
## Results for subgroups (fixed effect model):
##                k   MD        95%-CI    Q tau^2  I^2
## sub = Brass    3 0.01 [-0.49; 0.50] 1.29     0 0.0%
##
## Test for subgroup differences (fixed effect model):
##                    Q d.f. p-value
## Between groups 0.00    0       --
## Within groups  1.29    2  0.5248
##
## Results for subgroups (random effects model):
##                k   MD        95%-CI    Q tau^2  I^2
## sub = Brass    3 0.01 [-0.49; 0.50] 1.29     0 0.0%
##
## Test for subgroup differences (random effects model):
##                      Q d.f. p-value
```

```
## Between groups   0.00    0       --
##
## Details on meta-analytical method:
## - Inverse variance method
## - DerSimonian-Laird estimator for tau^2
```

END

Data END

```
## # A tibble: 7 x 10
##    ...1 author             year    Ne    Me    Se    Nc    Mc    Sc sub
##   <dbl> <chr>             <dbl> <dbl> <dbl> <dbl> <dbl> <dbl> <dbl> <chr>
## 1     1 Brattstrom SR ~  1989    20  3.79  1.28    10  3.28  0.92 Single r~
## 2     2 Herman SR         1981    41  2.92  1.82    18  2.65  0.95 Single r~
## 3     3 Pang SR           1976    21  3.81  1.07    15  4.8   1.88 Single r~
## 4     4 Brattstrom B b~  1989    18  3.19  0.99    20  4.11  0.81 Brass
## 5     5 Brattstrom B g~  1989    20  2.84  1.12    10  3.28  0.92 Brass
## 6     6 Herman B          1981    21  2.05  1.07    18  2.65  0.95 Brass
## 7     7 Pang B            1976    19  4.45  1.85    15  4.8   1.88 Brass
```

Plot END with subgroups random effects

Study or Subgroup	Windinstrument Mean	SD	Total	Control Mean	SD	Total	Weight	Mean Difference IV, Random, 95% CI
**Single reed**								
Brattstrom SR girls 1989	3.79	1.28	20	3.28	0.92	10	14.3%	0.51 [−0.29; 1.31]
Herman SR 1981	2.92	1.82	41	2.65	0.95	18	15.9%	0.27 [−0.44; 0.98]
Pang SR 1976	3.81	1.07	21	4.80	1.88	15	10.5%	−0.99 [−2.05; 0.07]
**Total (random effects, 95% CI)**			**82**			**43**	**40.7%**	**0.01 [−0.79; 0.80]**

Heterogeneity: $Tau^2 = 0.3059$; $Chi^2 = 5.32$, df = 2 (P = 0.07); $I^2 = 62\%$ [0%; 89%]

Test for overall effect: Z = 0.01 (P = 0.988)

Study or Subgroup	Windinstrument Mean	SD	Total	Control Mean	SD	Total	Weight	Mean Difference IV, Random, 95% CI
**Brass**								
Brattstrom B boys 1989	3.19	0.99	18	4.11	0.81	20	18.5%	−0.92 [−1.50; −0.34]
Brattstrom B girls 1989	2.84	1.12	20	3.28	0.92	10	15.1%	−0.44 [−1.19; 0.31]
Herman B 1981	2.05	1.07	21	2.65	0.95	18	17.4%	−0.60 [−1.23; 0.03]
Pang B 1976	4.45	1.85	19	4.80	1.88	15	8.3%	−0.35 [−1.61; 0.91]
**Total (random effects, 95% CI)**			**78**			**63**	**59.3%**	**−0.67 [−1.02; −0.31]**

Heterogeneity: $Tau^2 = 0$; $Chi^2 = 1.37$, df = 3 (P = 0.71); $I^2 = 0\%$ [0%; 66%]

Test for overall effect: Z = −3.66 (P < 0.001)

Study or Subgroup	Windinstrument Mean	SD	Total	Control Mean	SD	Total	Weight	Mean Difference IV, Random, 95% CI
**Total (random effects, 95% CI)**			**160**			**106**	**100.0%**	**−0.36 [−0.79; 0.08]**
**Prediction interval**								**[−1.59; 0.88]**

Heterogeneity: $Tau^2 = 0.1807$; $Chi^2 = 13.09$, df = 6 (P = 0.04); $I^2 = 54\%$ [0%; 80%]

Residual heterogeneity: $Tau^2 = NA$; $Chi^2 = 6.69$, df = 5 (P = 0.24); $I^2 = 25\%$ [0%; 69%]

Test for overall effect: Z = −1.61 (P = 0.107)

Test for subgroup differences: $Chi^2 = 2.28$, df = 1 (P = 0.13)

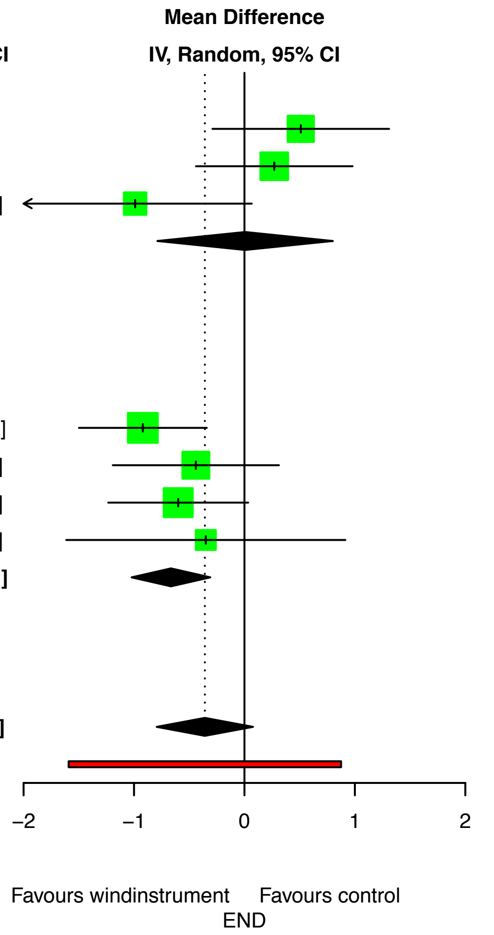

END analysis subgroup Single Reed

```
##                                MD         95%-CI %W(fixed) %W(random)
## Brattstrom SR girls 1989  0.51 [-0.29; 1.31]      35.1       34.8
## Herman SR 1981            0.27 [-0.44; 0.98]      44.7       37.6
## Pang SR 1976             -0.99 [-2.05; 0.07]      20.2       27.6
##
## Number of studies combined: k = 3
##
##                           MD         95%-CI    z p-value
## Fixed effect model      0.10 [-0.37; 0.57] 0.41  0.6788
## Random effects model 0.01 [-0.79; 0.80] 0.01  0.9882
## Prediction interval        [-8.71; 8.72]
##
## Quantifying heterogeneity:
## tau^2 = 0.3059; H = 1.63 [1.00; 3.05]; I^2 = 62.4% [0.0%; 89.3%]
##
## Test of heterogeneity:
##     Q d.f. p-value
##  5.32    2  0.0698
##
## Details on meta-analytical method:
## - Inverse variance method
## - DerSimonian-Laird estimator for tau^2
```

END analysis subgroup Brass

```
##                               MD          95%-CI %W(fixed) %W(random)
## Brattstrom B boys 1989  -0.92 [-1.50; -0.34]      37.9       37.9
## Brattstrom B girls 1989 -0.44 [-1.19;  0.31]      22.5       22.5
## Herman B 1981           -0.60 [-1.23;  0.03]      31.6       31.6
## Pang B 1976             -0.35 [-1.61;  0.91]       8.0        8.0
##
## Number of studies combined: k = 4
##
##                            MD          95%-CI     z p-value
## Fixed effect model   -0.67 [-1.02; -0.31] -3.66  0.0003
## Random effects model -0.67 [-1.02; -0.31] -3.66  0.0003
## Prediction interval         [-1.45;  0.12]
##
## Quantifying heterogeneity:
## tau^2 = 0; H = 1.00 [1.00; 1.73]; I^2 = 0.0% [0.0%; 66.4%]
##
## Test of heterogeneity:
##     Q d.f. p-value
##  1.37    3  0.7131
##
## Details on meta-analytical method:
## - Inverse variance method
## - DerSimonian-Laird estimator for tau^2
```

Plot END sensitivity analysis (without Brattstrom B girls)

Study or Subgroup	Windinstrument Mean	SD	Total	Control Mean	SD	Total	Weight	Mean Difference IV, Random, 95% CI
**Single reed**								
Brattstrom SR girls 1989	3.79	1.28	20	3.28	0.92	10	16.9%	0.51 [−0.29; 1.31]
Herman SR 1981	2.92	1.82	41	2.65	0.95	18	18.5%	0.27 [−0.44; 0.98]
Pang SR 1976	3.81	1.07	21	4.80	1.88	15	13.1%	−0.99 [−2.05; 0.07]
**Total (random effects, 95% CI)**			**82**			**43**	**48.6%**	**0.01 [−0.79; 0.80]**
Heterogeneity: $Tau^2 = 0.3059$; $Chi^2 = 5.32$, df = 2 (P = 0.07); $I^2 = 62\%$ [0%; 89%]								
Test for overall effect: Z = 0.01 (P = 0.99)								
**Brass**								
Brattstrom B boys 1989	3.19	0.99	18	4.11	0.81	20	20.9%	−0.92 [−1.50; −0.34]
Herman B 1981	2.05	1.07	21	2.65	0.95	18	19.9%	−0.60 [−1.23; 0.03]
Pang B 1976	4.45	1.85	19	4.80	1.88	15	10.6%	−0.35 [−1.61; 0.91]
**Total (random effects, 95% CI)**			**58**			**53**	**51.4%**	**−0.73 [−1.14; −0.33]**
Heterogeneity: $Tau^2 = 0$; $Chi^2 = 0.92$, df = 2 (P = 0.63); $I^2 = 0\%$ [0%; 77%]								
Test for overall effect: Z = −3.54 (P < 0.01)								
**Total (random effects, 95% CI)**			**140**			**96**	**100.0%**	**−0.34 [−0.86; 0.18]**
**Prediction interval**								**[−1.92; 1.23]**
Heterogeneity: $Tau^2 = 0.2503$; $Chi^2 = 13.07$, df = 5 (P = 0.02); $I^2 = 62\%$ [7%; 84%]								
Test for overall effect: Z = −1.29 (P = 0.20)								
Test for subgroup differences: $Chi^2 = 2.62$, df = 1 (P = 0.11)								

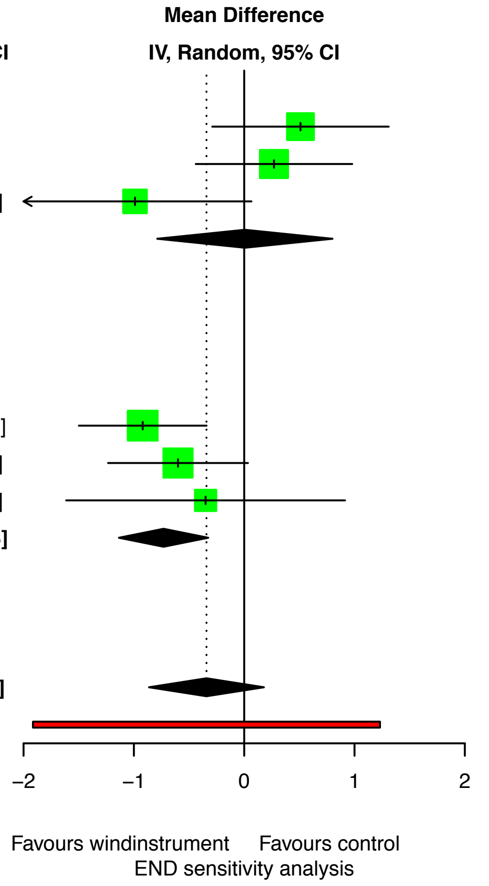

END sensitivity analysis subgroup Brass

```
##                            MD          95%-CI %W(fixed) %W(random)   sub
## Brattstrom B boys 1989 -0.92 [-1.50; -0.34]      48.9       48.9 Brass
## Herman B 1981          -0.60 [-1.23;  0.03]      40.8       40.8 Brass
## Pang B 1976            -0.35 [-1.61;  0.91]      10.3       10.3 Brass
##
## Number of studies combined: k = 3
##
##                          MD          95%-CI     z p-value
## Fixed effect model    -0.73 [-1.14; -0.33] -3.54  0.0004
## Random effects model -0.73 [-1.14; -0.33] -3.54  0.0004
## Prediction interval         [-3.36;  1.89]
##
## Quantifying heterogeneity:
## tau^2 = 0; H = 1.00 [1.00; 2.11]; I^2 = 0.0% [0.0%; 77.4%]
##
## Quantifying residual heterogeneity:
## H = 1.00 [1.00; 2.11]; I^2 = 0.0% [0.0%; 77.4%]
##
## Test of heterogeneity:
##     Q d.f. p-value
##  0.92    2  0.6305
##
## Results for subgroups (fixed effect model):
##               k    MD          95%-CI    Q tau^2  I^2
## sub = Brass   3 -0.73 [-1.14; -0.33] 0.92     0 0.0%
##
## Test for subgroup differences (fixed effect model):
##                    Q d.f. p-value
## Between groups 0.00    0      --
## Within groups  0.92    2  0.6305
##
## Results for subgroups (random effects model):
##               k    MD          95%-CI    Q tau^2  I^2
## sub = Brass   3 -0.73 [-1.14; -0.33] 0.92     0 0.0%
##
## Test for subgroup differences (random effects model):
##                      Q d.f. p-value
```

END sensitivity analysis subgroup Brass

```
## Between groups   0.00    0      --
##
## Details on meta-analytical method:
## - Inverse variance method
## - DerSimonian-Laird estimator for tau^2
```

DIFFERENCE

Data DIFFERENCE

```
## # A tibble: 7 x 10
##    ...1 author            year    Ne     Me    Se    Nc    Mc    Sc sub
##   <dbl> <chr>            <dbl> <dbl>  <dbl> <dbl> <dbl> <dbl> <dbl> <chr>
## 1     1 Brattstrom SR ~  1989    20 -0.74   0.88    10 -0.2   0.23 Single ~
## 2     2 Herman SR        1981    41  0.42   1.73    18 -0.11  1.19 Single ~
## 3     3 Pang SR          1976    21 -0.18   0.96    15 -0.08  0.92 Single ~
## 4     4 Brattstrom B b~  1989    18 -0.64   0.72    20  0.06  0.07 Brass
## 5     5 Brattstrom B g~  1989    20 -0.290  0.45    10 -0.2   0.23 Brass
## 6     6 Herman B         1981    21 -0.75   1.39    18 -0.11  1.19 Brass
## 7     7 Pang B           1976    19 -0.96   1.26    15 -0.08  0.92 Brass
```

Plot DIFFERENCE with subgroups random effects

Study or Subgroup	Windinstrument Mean	SD	Total	Control Mean	SD	Total	Weight	Mean Difference IV, Random, 95% CI
**Single reed**								
Brattstrom SR girls 1989	−0.74	0.88	20	−0.20	0.23	10	17.2%	−0.54 [−0.95; −0.13]
Herman SR 1981	0.42	1.73	41	−0.11	1.19	18	10.0%	0.53 [−0.23; 1.29]
Pang SR 1976	−0.18	0.96	21	−0.08	0.92	15	12.5%	−0.10 [−0.72; 0.52]
**Total (random effects, 95% CI)**			**82**			**43**	**39.7%**	**−0.10 [−0.70; 0.49]**
Heterogeneity: $Tau^2 = 0.1842$; $Chi^2 = 6.15$, df = 2 (P = 0.05); $I^2 = 67\%$ [0%; 91%]								
Test for overall effect: Z = −0.35 (P = 0.730)								
**Brass**								
Brattstrom B boys 1989	−0.64	0.72	18	0.06	0.07	20	19.2%	−0.70 [−1.03; −0.37]
Brattstrom B girls 1989	−0.29	0.45	20	−0.20	0.23	10	21.4%	−0.09 [−0.33; 0.15]
Herman B 1981	−0.75	1.39	21	−0.11	1.19	18	9.3%	−0.64 [−1.45; 0.17]
Pang B 1976	−0.96	1.26	19	−0.08	0.92	15	10.5%	−0.88 [−1.61; −0.15]
**Total (random effects, 95% CI)**			**78**			**63**	**60.3%**	**−0.51 [−0.95; −0.08]**
Heterogeneity: $Tau^2 = 0.1275$; $Chi^2 = 11.1$, df = 3 (P = 0.01); $I^2 = 73\%$ [24%; 90%]								
Test for overall effect: Z = −2.33 (P = 0.020)								
**Total (random effects, 95% CI)**			**160**			**106**	**100.0%**	**−0.36 [−0.67; −0.04]**
**Prediction interval**								**[−1.28; 0.57]**

Heterogeneity: $Tau^2 = 0.1042$; $Chi^2 = 17.58$, df = 6 ($P < 0.01$); $I^2 = 66\%$ [24%; 85%]

Residual heterogeneity: Tau^2 = NA; $Chi^2 = 17.24$, df = 5 ($P < 0.01$); $I^2 = 71\%$ [33%; 88%]

Test for overall effect: Z = −2.24 (P = 0.025)

Test for subgroup differences: $Chi^2 = 1.19$, df = 1 (P = 0.27)

Mean Difference IV, Random, 95% CI

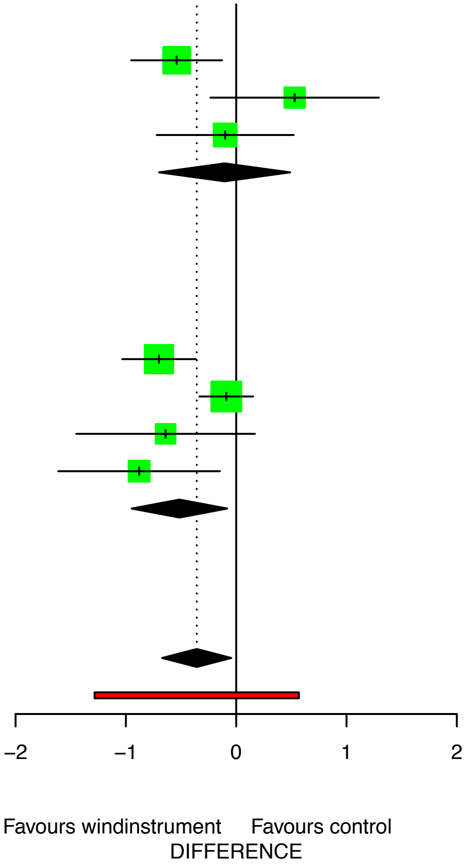

DIFFERENCE analysis subgroup Single Reed

```
##                               MD         95%-CI %W(fixed) %W(random)
## Brattstrom SR girls 1989 -0.54 [-0.95; -0.13]      57.8       40.3
## Herman SR 1981            0.53 [-0.23;  1.29]      16.8       27.4
## Pang SR 1976             -0.10 [-0.72;  0.52]      25.4       32.3
##
## Number of studies combined: k = 3
##
##                           MD        95%-CI     z p-value
## Fixed effect model   -0.25 [-0.56; 0.06] -1.56  0.1190
## Random effects model -0.10 [-0.70; 0.49] -0.35  0.7297
## Prediction interval        [-6.78; 6.57]
##
## Quantifying heterogeneity:
## tau^2 = 0.1842; H = 1.75 [1.00; 3.26]; I^2 = 67.5% [0.0%; 90.6%]
##
## Test of heterogeneity:
##     Q d.f. p-value
##  6.15    2  0.0463
##
## Details on meta-analytical method:
## - Inverse variance method
## - DerSimonian-Laird estimator for tau^2
```

DIFFERENCE analysis subgroup Brass

```
##                              MD          95%-CI %W(fixed) %W(random)
## Brattstrom B boys 1989  -0.70 [-1.03; -0.37]      30.7       31.2
## Brattstrom B girls 1989 -0.09 [-0.33;  0.15]      57.8       34.2
## Herman B 1981           -0.64 [-1.45;  0.17]       5.2       16.4
## Pang B 1976             -0.88 [-1.61; -0.15]       6.4       18.3
##
## Number of studies combined: k = 4
##
##                          MD          95%-CI     z p-value
## Fixed effect model   -0.36 [-0.54; -0.17] -3.77  0.0002
## Random effects model -0.51 [-0.95; -0.08] -2.33  0.0199
## Prediction interval        [-2.32;  1.29]
##
## Quantifying heterogeneity:
## tau^2 = 0.1275; H = 1.92 [1.15; 3.23]; I^2 = 73.0% [23.8%; 90.4%]
##
## Test of heterogeneity:
##      Q d.f. p-value
##  11.10    3  0.0112
##
## Details on meta-analytical method:
## - Inverse variance method
## - DerSimonian-Laird estimator for tau^2
```

Plot DIFFERENCE sensitivity analysis (without Brattstrom B girls)

Study or Subgroup	Windinstrument Mean	SD	Total	Control Mean	SD	Total	Weight	Mean Difference IV, Random, 95% CI
**Single reed**								
Brattstrom SR girls 1989	−0.74	0.88	20	−0.20	0.23	10	22.2%	−0.54 [−0.95; −0.13]
Herman SR 1981	0.42	1.73	41	−0.11	1.19	18	12.5%	0.53 [−0.23; 1.29]
Pang SR 1976	−0.18	0.96	21	−0.08	0.92	15	15.8%	−0.10 [−0.72; 0.52]
**Total (random effects, 95% CI)**			**82**			**43**	**50.4%**	**−0.10 [−0.70; 0.49]**
Heterogeneity: $Tau^2 = 0.1842$; $Chi^2 = 6.15$, df = 2 (P = 0.05); $I^2 = 67\%$ [0%; 91%]								
Test for overall effect: Z = −0.35 (P = 0.73)								
**Brass**								
Brattstrom B boys 1989	−0.64	0.72	18	0.06	0.07	20	24.8%	−0.70 [−1.03; −0.37]
Herman B 1981	−0.75	1.39	21	−0.11	1.19	18	11.6%	−0.64 [−1.45; 0.17]
Pang B 1976	−0.96	1.26	19	−0.08	0.92	15	13.1%	−0.88 [−1.61; −0.15]
**Total (random effects, 95% CI)**			**58**			**53**	**49.6%**	**−0.72 [−1.00; −0.44]**
Heterogeneity: $Tau^2 = 0$; $Chi^2 = 0.23$, df = 2 (P = 0.89); $I^2 = 0\%$ [0%; 11%]								
Test for overall effect: Z = −4.96 (P < 0.01)								
**Total (random effects, 95% CI)**			**140**			**96**	**100.0%**	**−0.43 [−0.78; −0.09]**
**Prediction interval**								**[−1.42; 0.55]**
Heterogeneity: $Tau^2 = 0.0953$; $Chi^2 = 11.15$, df = 5 (P = 0.05); $I^2 = 55\%$ [0%; 82%]								
Test for overall effect: Z = −2.46 (P = 0.01)								
Test for subgroup differences: $Chi^2 = 3.34$, df = 1 (P = 0.07)								

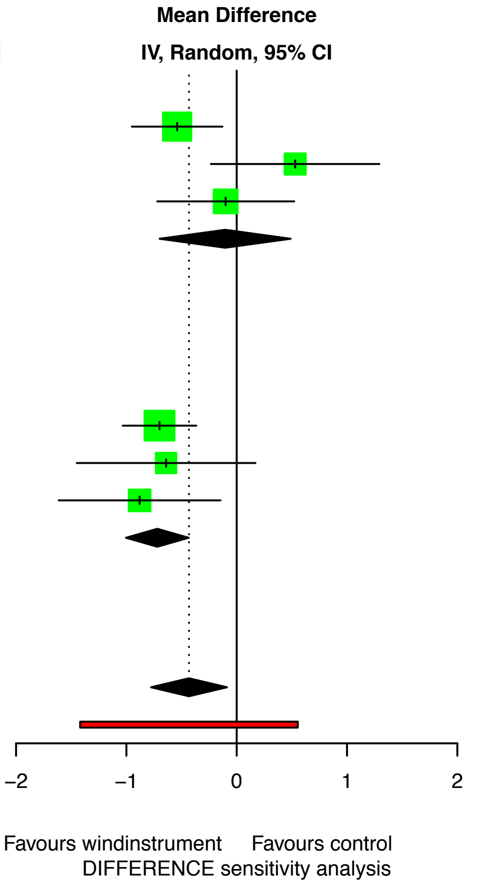

DIFFERENCE sensitivity analysis subgroup Brass

```
##                           MD          95%-CI %W(fixed) %W(random)   sub
## Brattstrom B boys 1989 -0.70 [-1.03; -0.37]      72.6       72.6 Brass
## Herman B 1981          -0.64 [-1.45;  0.17]      12.4       12.4 Brass
## Pang B 1976            -0.88 [-1.61; -0.15]      15.1       15.1 Brass
##
## Number of studies combined: k = 3
##
##                         MD          95%-CI     z  p-value
## Fixed effect model   -0.72 [-1.00; -0.44] -4.96 < 0.0001
## Random effects model -0.72 [-1.00; -0.44] -4.96 < 0.0001
## Prediction interval        [-2.56;  1.13]
##
## Quantifying heterogeneity:
## tau^2 = 0; H = 1.00 [1.00; 1.06]; I^2 = 0.0% [0.0%; 11.1%]
##
## Quantifying residual heterogeneity:
## H = 1.00 [1.00; 1.06]; I^2 = 0.0% [0.0%; 11.1%]
##
## Test of heterogeneity:
##     Q d.f. p-value
##  0.23    2  0.8895
##
## Results for subgroups (fixed effect model):
##               k    MD          95%-CI    Q tau^2  I^2
## sub = Brass   3 -0.72 [-1.00; -0.44] 0.23     0 0.0%
##
## Test for subgroup differences (fixed effect model):
##                     Q d.f. p-value
## Between groups 0.00    0      --
## Within groups  0.23    2  0.8895
##
## Results for subgroups (random effects model):
##               k    MD          95%-CI    Q tau^2  I^2
## sub = Brass   3 -0.72 [-1.00; -0.44] 0.23     0 0.0%
##
## Test for subgroup differences (random effects model):
##                       Q d.f. p-value
```

DIFFERENCE sensitivity analysis subgroup Brass

```
## Between groups   0.00    0       --
##
## Details on meta-analytical method:
## - Inverse variance method
## - DerSimonian-Laird estimator for tau^2
```

References

(Schwarzer et al. 2015) (Schwarzer 2007) (Viechtbauer 2010) (IntHout et al. 2016)

IntHout J, Ioannidis JP, Rovers MM, Goeman JJ. 2016. Plea for routinely presenting prediction intervals in meta-analysis. BMJ open. 6(7):e010247.

Schwarzer G. 2007. Meta: An r package for meta-analysis. R news. 7(3):40–45.

Schwarzer G, Carpenter JR, Rücker G. 2015. Meta-analysis with r. Springer.

Viechtbauer W. 2010. Conducting meta-analyses in r with the metafor package. Journal of Statistical Software. 36(3):1–48.
